# Integrative mRNA and miRNA Profiling Identifies Shared and Subtype-Associated PI3K–AKT–mTOR Pathway Dysregulation and Candidate miRNA-Mediated Regulatory Interactions in Endometriosis-Associated Ovarian Cancers (EAOCs)

**DOI:** 10.3390/biomedicines14091991

**Published:** 2026-09-04

**Authors:** Radwa Hablase, Cristina Sisu, Sayeh Saravi, Suzana Panfilov, Emmanouil Karteris, Jayanta Chatterjee

**Affiliations:** 1 College of Health, Medicine and Life Sciences, Brunel University London, Uxbridge UB8 3PH, UK; cristina.sisu@brunel.ac.uk (C.S.); sayeh.saravi@brunel.ac.uk (S.S.); suzana.panfilov2@brunel.ac.uk (S.P.); emmanouil.karteris@brunel.ac.uk (E.K.); jayanta.chatterjee1@nhs.net (J.C.); 2 Academic Department of Gynaecological Oncology, Royal Surrey NHS Foundation Trust Hospital, Guildford GU2 7XX, UK

**Keywords:** ovarian cancer, mTOR, TCGA, GTEx, miRNA

## Abstract

**Background:** Endometriosis-associated ovarian cancers (EAOCs), including ovarian clear cell (OCCC) and endometrioid ovarian carcinoma (EnOC) subtypes, frequently exhibit transcriptomic dysregulation of the PI3K/AKT/mTOR signalling axis. While genomic aberrations are well-documented, the coordinated microRNA (miRNA)-mediated networks governing post-transcriptional remodelling of this pathway across these subtypes remain poorly defined. **Methods:** We conducted an integrative in silico meta-analysis of independent mRNA and small RNA sequencing datasets. The mRNA analysis included 120 EAOC samples, of which 68 were OCCC and 52 were EnOC, compared with 149 normal ovarian tissues. The miRNA analysis included 170 samples comprising 55 OCCC, 82 EnOC and 33 normal ovarian tissues. Differential expression analysis, dimensionality reduction (UMAP), functional enrichment, and topologically unweighted miRNA–mRNA interaction networks were evaluated. **Results:** Both subtypes showed significant transcriptomic dysregulation of the core pathway machinery, including *PIK3CB* and *mTOR*, while preserving mTORC2 components. Post-transcriptional concurrent downregulation of *IRS1, GRB10, DDIT4*, and *PIK3CD*, which were identified as candidate targets of the hub miRNAs hsa-miR-30a-5p, hsa-miR-30d-5p, and hsa-miR-7-5p, suggests further refined control of the pathway. The identification of highly connected hub genes linking mTOR, MAPK, and Wnt signalling pathways within the mTOR-regulatory network suggests that pathway modulation occurs through extensive crosstalk across multiple oncogenic signalling pathways in EAOCs. **Conclusions:** Transcriptomic dysregulation of the mTOR pathway in EAOCs reflects not only genomic alterations but also potential post-transcriptional regulation. Despite subtype-specific transcriptomic differences, both exhibited transcriptional upregulation of components of the canonical PI3K/AKT/mTOR signalling axis. Pathway modulation through the miRNA regulatory network exhibited potential crosstalk across oncogenic pathways and hub genes.

## 1. Introduction

Endometriosis-associated ovarian cancers (EAOCs) represent a distinct subgroup of epithelial ovarian malignancies that arise in the context of endometriosis. The term includes ovarian clear cell carcinoma (OCCC) and endometrioid ovarian carcinoma (EnOC) [1,2]. Together, they represent the second most common subtype of ovarian carcinomas after the high-grade serous ovarian cancer (HGSOC) group and account for approximately 10–20% of all epithelial ovarian cancers. The prevalence differs geographically, with the highest incidence observed in East Asian populations, where it can reach 25–30% [3].

Clinically, EAOC patients tend to present at an earlier stage and are generally younger compared to HGSOC patients. The prognosis is overall better. However, advanced-stage and recurrent diseases are often chemo-resistant and carry a worse prognosis than HGSOC [4]. Such differences in biological behaviour dictate a subtype-specific approach when investigating the molecular mechanisms underlying the disease.

The mechanistic target of rapamycin (mTOR) is a serine/threonine protein kinase belonging to the phosphatidylinositol 3-kinase-related kinase (PIKK) family. It exists in two functionally distinct complexes, the mechanistic target of rapamycin complex 1 (mTORC1) and the mechanistic target of rapamycin complex 2 (mTORC2), and is regarded as the central regulator of cellular homeostasis, balancing anabolic and catabolic processes in response to external and internal cues [2,5].

Previously, we investigated the mTOR pathway dysregulation in chemotherapy-naive high-grade serous primary ovarian cancers using transcriptomic and microRNA (miRNA) sequencing data from The Cancer Genome Atlas (TCGA) and Genotype-Tissue Expression (GTEx) consortia projects [5].

To model the mTOR pathway dysregulation in ovarian clear cell (OCCC) and endometrioid ovarian carcinoma (EnOC), we adopted an integrative approach, examining multiple dependent datasets to achieve sufficient sample size. A gene expression meta-analysis approach was adopted, considering that these two histological subtypes are not represented in the TCGA ovarian cancer cohort. Given their relatively low prevalence, the integrative approach facilitated evaluation of the transcriptional alterations.

## 2. Materials and Methods

### 2.1. Datasets and Sample Selection

To identify mRNA data sets, the Gene Expression Omnibus (GEO) repository (GEO Accession viewer) was searched using the following MeSH terms and free-text keywords: ((((“ovarian neoplasms”[All Fields] OR “ovarian cancer”[All Fields] OR “ovarian carcinoma”[All Fields]))) OR “ovarian neoplasms”[MeSH Terms]) AND ((“ovarian clear cell carcinoma”[All Fields] OR “clear cell ovarian carcinoma”[All Fields] OR “OCCC”[All Fields] OR “endometrioid carcinoma”[All Fields] OR “endometrioid adenocarcinoma”[All Fields] OR “ENOC”[All Fields] OR “endometriosis associated ovarian carcinoma”[All Fields] OR “EAOC”[All Fields])). The filters “Homo sapiens” and “Expression profiling by high-throughput sequencing” were then applied.

The initial search yielded 48 GEO datasets. Each dataset was manually screened, and selection was limited to datasets that included ovarian clear cell and/or endometrioid ovarian carcinoma samples from patients. Cell lines, xenografted models, single-cell RNA sequencing, and datasets based on blood-, serum-, or urine-derived biomarkers (e.g., platelet RNA profiles) were excluded. Additionally, datasets in which EAOCs were not clearly stratified into endometrioid or clear cell were excluded (Supplementary Table S1).

Each dataset was further examined to include only samples with clear annotations as ovarian clear cell or endometrioid ovarian carcinoma across all stages and grades. Where neoadjuvant chemotherapy, interval debulking surgery, refractory or resistant were explicitly mentioned, or sample-level data could not be obtained with certainty, datasets were excluded. Datasets with a single sample or mixed histology were also removed.

Normal ovarian samples were obtained from the Genotype-Tissue Expression (GTEx) (https://www.gtexportal.org, accessed on 5 May 2026) project.

For miRNA data, the Gene Expression Omnibus (GEO) repository (https://ncbi-nlm-nih-gov/geo/, accessed on 5 May 2026) was queried using the following free-text keywords: (ovary) AND cancer. The filters “Homo sapiens” and “Non-coding RNA profiling by high-throughput sequencing” were then applied. The initial search identified 43 datasets. The same inclusion and exclusion criteria used for the mRNA datasets were applied to the miRNA dataset selection. The identified GEO datasets included normal ovarian tissue samples, which were used as controls. The GEO datasets comprised high-throughput, open-access miRNA expression profiles for ovarian clear cell carcinoma. However, no comparable datasets were available for EnOC. (Supplementary Table S2).

ArrayExpress was then searched using the keywords “ovarian adenocarcinoma,” “ovarian cancer,” “ovarian clear cell adenocarcinoma,” and “endometrioid adenocarcinoma of the ovary”. The following filters were applied: Homo sapiens, high-throughput sequencing, RNA-seq of coding RNA, RNA-seq of non-coding RNA, and technology: sequencing array. The search was further limited to studies with available raw datasets. After applying these criteria, no additional studies met the predefined inclusion and exclusion criteria. The European Genome-Phenome Archive was searched using the keyword “miRNA”; 156 studies were screened. Access was requested and granted for dataset EGAD00001009636, which contains miRNA data from ovarian clear cell and endometrioid carcinomas [6]. Sample identifiers were cross-checked across mRNA and miRNA datasets, and no duplicate biological samples were identified. All datasets included in the mRNA and miRNA analyses were independent, except for GSE230956, which contributed data to both analyses. (Supplementary Tables S3–S5).

**Figure 1:**
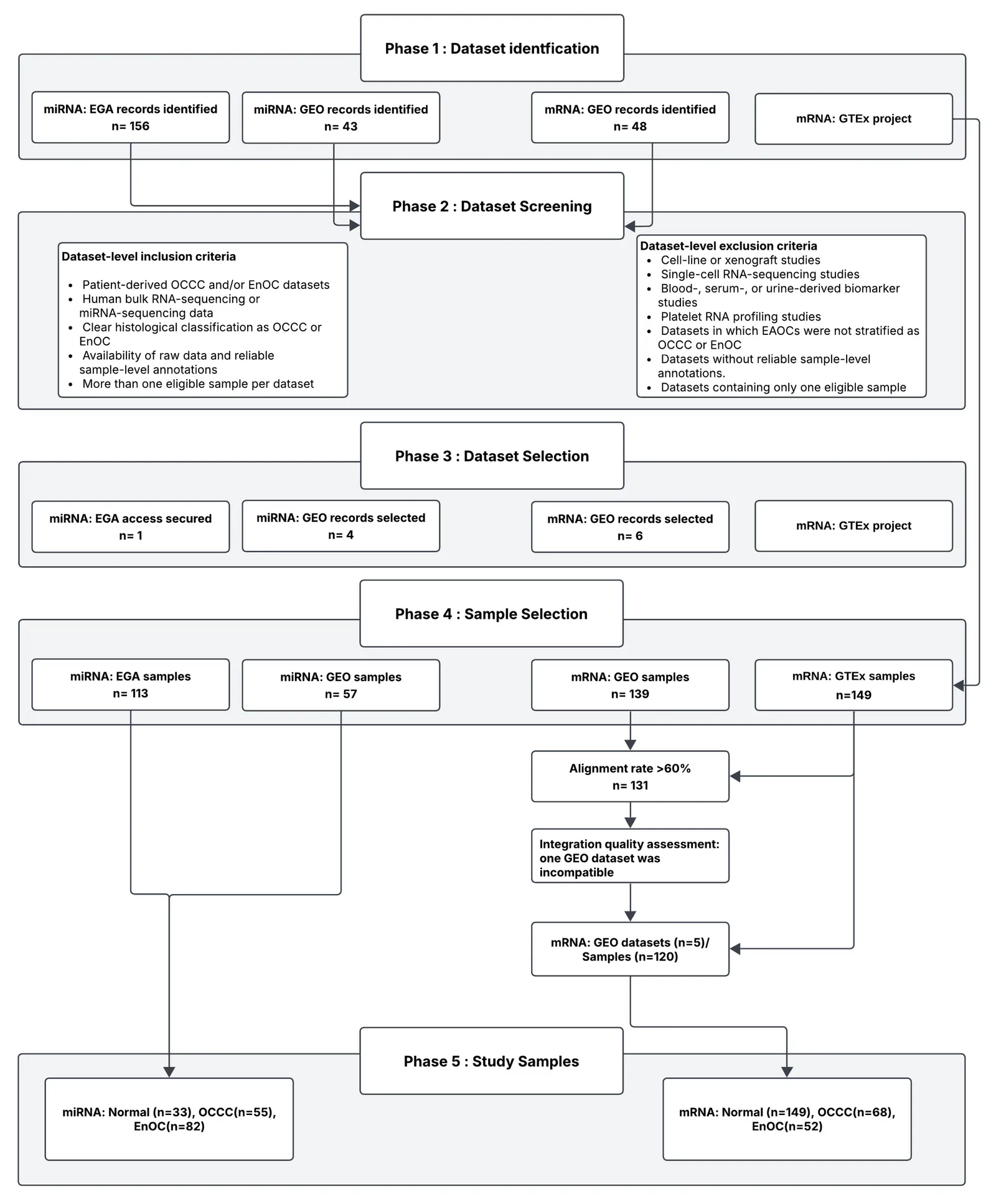
Flow diagram of dataset identification, screening, selection and final sample composition for the mRNA and miRNA analyses. GEO, EGA and GTEx records were screened according to predefined dataset- and sample-level eligibility criteria. For the mRNA analysis, six GEO datasets comprising 139 cancer samples were selected. Eight samples with less than 60% read alignment were excluded, and technical sequencing runs from the same biological samples in GSE230956 were collapsed before count-matrix construction, leaving 131 samples. Following integration and quality assessment, GSE160692 was excluded, resulting in a final cancer cohort of 120 samples from five GEO datasets. These samples were analysed alongside 149 normal ovarian tissue samples from GTEx. For the miRNA analysis, four GEO datasets and one EGA dataset were included, yielding 170 samples: 55 OCCC, 82 EnOC and 33 normal ovarian tissue samples. EGA, European Genome-Phenome Archive; EnOC, endometrioid ovarian carcinoma; GEO, Gene Expression Omnibus; GTEx, Genotype-Tissue Expression; OCCC, ovarian clear cell carcinoma.

For mRNA analysis, six GEO datasets, GSE230956, GSE189553, GSE226870, GSE121103, GSE295399 and GSE160692 were initially identified, including 139 samples (87 OCCC + 52 EnOC). In the data processing step, eight samples with <60% alignment were excluded (Supplementary Table S6). Additionally, the dataset GSE230956 contained two single-end runs from the same mRNA library for each biological sample, which were collapsed into one sample before creating the count matrices, leaving six datasets and 131 samples (79 OCCC + 52 EnOC). Following batch correction and normalisation, the dataset GSE160692 was excluded from further analysis, and the final study cancer cohort included five GEO datasets and 120 samples (68 OCCC + 52 EnOC) (Table 1). The GTEx normal tissue cohort included 149 samples. The dataset identification, screening and sample-selection processes for the mRNA and miRNA analyses are summarised in Figure 1.

For miRNA analysis, four GEO and one European Genome-Phenome.

Archive datasets were included in the final analysis. The total number of samples included was 170: 82 EnOC, 55 OCCC, and 33 normal ovarian tissues (Table 2).

### 2.2. Data Processing

We downloaded mRNA data using the prefetch function of sratoolkit (v3.0.7), then used fastq-dump (2.11.3) to extract the fastq files. Raw data from all datasets were quality-assessed using FastQC (v0.11.9); two datasets were identified as containing poor-quality bases and adaptor sequences, which were trimmed using Fastp (v0.24.0). The fastp parameters used for the dataset GSE160692 were (*-i -I -o -O -w -h -j -- low_complexity_filter – complexity threshold 30*). In addition, the fastp parameters used for the dataset GSE295399 were (*--cut_front --cut_front_mean_quality 20 --cut_front_window_size 4 --cut_tail --cut_tail_mean_quality 20 --cut_tail_window_size 4 --qualified_quality_phred 20 --unqualified_percent_limit 40 --trim_front1 3 --trim_front2 3*).

We used the human reference genome GRCh38 (GENCODE release v39) to generate the genome index for read alignment using STAR (v2.7.10a). We used GENCODE v39 as the reference annotation. Reads were aligned to the reference genome using STAR (v2.7.10a), and gene-level expected counts were quantified using RSEM (v1.3.3), consistent with the GTEx RNA-seq processing pipeline [7,8]. Samples with a total alignment percentage < 60% were excluded. Gene-level read counts from RSEM v.1.3.3 were imported into R (v 4.4.1) using the tximport package (v1.34.0).

The GTEx Transcript read counts from RSEM v.1.3.3 (v 10) of normal ovarian samples were downloaded from the GTEx portal and collapsed to gene-level reads.

For the miRNA datasets, raw sequencing data were downloaded using the prefetch function of sratoolkit (v3.0.7), and then we used fastq-dump (2.11.3) to extract the fastq files. For the EGA dataset, we downloaded the raw sequence data as fastq files using the pyega3 client downloader (v5.2.0). Raw sequencing reads were assessed for quality using fastqc (v0.11.9). Adapter sequences were removed using fastp (v0.24.0) for the GEO datasets and cutadapt (v 1.18) for the EGA dataset. We used the human reference genome GRCh38 (GENCODE release 49) and generated a genome index with Bowtie2 (v2.4.2). Small RNA sequencing reads in the fastq format were aligned to the GRCh38 reference genome using Bowtie, applying the following parameters (*-n 2 -l 15 -k 1*). Alignment outputs were converted to sorted BAM files using samtools (v1.12). miRNA read quantification was performed using featureCounts of subread (v2.0.2) with the miRBase (release 22.1) GFF3 annotation file (hsa. gff3). The resulting count tables were subsequently combined into an miRNA count matrix for downstream analysis.

### 2.3. Data Integration and Normalisation

For samples included in the mRNA analysis, low-abundance transcripts were filtered by retaining only those with a minimum of 5 read counts in at least 10% of samples.

Batch correction within each data source, GEO and GTEx, was performed using ComBat-seq from the sva package in R (v3.54.0). For the GEO cancer cohort, the study identifier was used as the batch variable. For the GTEx dataset, the sequencing centre identifier was used as the batch variable.

The two matrices were then merged by Ensembl gene identifiers and log-transformed. Quantile normalisation was performed on the combined expression matrix using the *normalise.quantiles* function from the preprocessCore R package (v1.68.0) to ensure a uniform distribution across samples and reduce the impact of heteroscedasticity [9]. Data structure, distributional comparability and residual cross-study technical heterogeneity were evaluated before and after batch correction and normalisation using principal component analysis (PCA), relative log expression (RLE) plots and expression-density plots. PCA was performed using prcomp from the R stats package (v4.4.1), visualised using ggplot2 (v 4.0.2), and stratified by dataset, sequencing platform, sequencing centre, histological subtype and disease status. RLE values and boxplots were generated using base R functions, while expression-density data were reshaped using reshape2 (v1.4.4) and visualised using ggplot2 (v4.0.2) R packages (Supplementary Material File S7).

For miRNA analysis, differential expression analysis was performed using DESeq2, with the recorded dataset/batch and disease condition included in the negative binomial model (~ batch + Condition) to account for technical variation. EAOC samples were compared with normal ovarian tissue. Low-abundance miRNAs were filtered, retaining only those with read counts of more than 5 in at least 10% of the samples. Principal component analysis (PCA) was performed on variance-stabilised miRNA expression values generated using DESeq2 (v1.46.0). For visualisation, dataset-associated batch effects were removed using the removeBatchEffect function in the limma package (v3.62.2) while preserving cancer-versus-normal differences. PCA was conducted on the 500 most variable miRNAs, and the samples were visualised according to histotype and dataset of origin.

### 2.4. Differential Expression Analysis

For the mRNA analysis, differential gene expression analysis between the normal and pooled EAOC samples was conducted using the LIMMA package (v3.62.2) in Bioconductor (https://www.bioconductor.org/, accessed on 5 May 2026). The design formula utilised was the ~ 0 + condition to compute the contrast between EAOC and normal ovarian tissues. Crucially, to account for technical variance across different cohorts, we utilised the duplicateCorrelation function, setting the source dataset as a blocking variable to model intra-batch correlation as a random effect. Clinical covariates (stage, grade, treatment) were omitted from the model because harmonised, complete metadata was not uniformly available across all aggregated GEO cohorts. Multiple testing was corrected using the Benjamini–Hochberg false-discovery rate (FDR) method. Significantly dysregulated genes were defined as having an adjusted *p*-value < 0.05 and a log2FC (fold change) >1 or <−1 [10]. (Supplementary Table S8).

Leave-one-dataset-out sensitivity analysis was initially performed across the six GEO cancer datasets to assess the influence of individual studies on the pooled results. Each dataset was excluded in turn, and the differential-expression model was refitted using the same limma design as the corresponding pooled analysis. These results were assessed alongside a principal component analysis, relative log expression, and expression-density plots to evaluate each dataset’s suitability for integration. GSE160692 was subsequently excluded because of persistent dataset-specific expression patterns, and the sensitivity analysis was repeated across the five retained datasets. Results from each iteration were compared with those of the corresponding complete cohort using the number and overlap of significantly differentially expressed genes, Spearman correlations of log2 fold changes, and concordance in the direction of expression changes. Differential-expression models were fitted using limma (v3.62.2). Correlations and directional concordance were calculated using base R functions, and the results were summarised using dplyr (v1.1.4) (Supplementary Material File S7, Supplementary Tables S9 and S10).

A study-level random-effects meta-analysis was performed as a sensitivity analysis of the primary pooled differential-expression results. Five Gene Expression Omnibus cancer (GEO) datasets (GSE121103, GSE189553, GSE226870, GSE230956, and GSE295399) were analysed against a shared reference comprising 149 normal ovarian samples from the Genotype-Tissue Expression project (GTEx). The analysis used the previously filtered gene-count matrix before ComBat-seq correction and quantile normalisation. Library sizes were normalised using the trimmed mean of the M-values method in edgeR (v4.4.2), followed by voom transformation and precision weighting in limma (v3.62.2). A linear model was fitted with separate coefficients for each GEO dataset and each GTEx sequencing centre. For each cancer dataset, differential expression was estimated relative to an equally weighted reference derived from the two GTEx sequencing centres, and empirical Bayes moderation was applied.

Because all five cancer datasets were compared with the same normal samples, their effect estimates were statistically dependent. Gene-specific covariance arising from the shared controls was estimated from the voom precision weights and limma posterior residual variances. A decoupling transformation was then applied to account for this dependence in the study-level variances [11]. The adjusted dataset-specific estimates were combined using a random-effects model with restricted maximum-likelihood estimation in metafor (v5.0.1). The genes for which the model could not be estimated were excluded. The *p*-values were adjusted using the Benjamini–Hochberg method, and genes with an adjusted *p*-value < 0.05 and an absolute pooled log_2_ fold change > 1 were considered significantly differentially expressed.

Agreement between the sensitivity meta-analysis and the primary pooled EAOC analysis was evaluated using Spearman rank correlation and directional concordance. Differentially expressed genes identified in the primary analysis were considered supported when they showed the same direction of regulation and met both the adjusted *p*-value and fold-change thresholds in the meta-analysis. Directional consistency across the five individual cancer datasets was also assessed. Between-dataset heterogeneity was summarised using τ^2^ and I^2^. I^2^ values were classified as low (<25%), moderate (25–<50%), substantial (50–<75%), or high (≥75%). Given the use of shared controls and the limited number of datasets, the heterogeneity estimates were interpreted descriptively (Supplementary Tables S11 and S12).

Differential expression analysis of the miRNA in EAOC samples versus normal ovarian tissues was performed in R using the DESeq2 package (v1.46.0) [12]. DESeq2 internally estimates sample-specific size factors for normalisation and gene-wise dispersion parameters. For the overall EAOC-versus-normal comparison, the DESeq2 design included the disease status (Condition) and the data source (batch) as model terms (design = ~ Condition + batch), using the cancer-versus-normal contrast. By default, DESeq2 adjusts the *p*-values using the Benjamini–Hochberg (BH) method. Statistical significance was defined using a threshold of an adjusted *p*-value < 0.05 and an absolute log2 fold change > 1 (Supplementary Table S13).

Leave-one-dataset-out (LODO) sensitivity analyses were performed by sequentially excluding each miRNA dataset and repeating the same DESeq2 analysis used in the pooled analysis, using a model adjusted for batch and disease status. Robustness was assessed by comparing log_2_ fold changes between the full and reduced models using Spearman’s correlation and directional concordance. Reduced models were not fitted when exclusion of a dataset resulted in a rank-deficient design that prevented separation of the batch and disease effects. The number of significantly differentially expressed miRNAs following each dataset exclusion and the numbers of cancer and normal samples were also recorded for each iteration to facilitate interpretation of the changes arising from dataset exclusion. Differential-expression models were fitted using the DESeq2 package in R (v1.46.0), Spearman correlations and effect–direction concordance were calculated using base R functions, and summary results were assembled using the dplyr package in R (v 1.1.4) (Supplementary Table S14).

### 2.5. Comparative Transcriptomic Analysis Between Endometriosis-Associated Ovarian Cancer Subtypes

To assess shared and subtype-specific transcriptional changes between endometrioid ovarian carcinoma and ovarian clear cell carcinoma, differential gene expression analysis was performed separately for each histological subtype relative to normal ovarian tissue using the limma package (v3.62.2) and the same model design and significance thresholds applied in the pooled analysis. (Supplementary Tables S15 and S16). Overlap analysis was performed using the sets of significantly differentially expressed genes from each group. Venn diagrams were generated to visualise the number of unique and overlapping genes using the ggVennDiagram (1.5.7) package of R (Supplementary Material File S7, Figure S7). Effect–size concordance of the overlapping genes was assessed using Spearman correlation of log2 fold changes, and directional concordance was calculated. This was performed using the R stats package (v 4.4.1), and plot visualisation using the ggplot2 package (v 4.0.2). Gene variance across endometrioid ovarian carcinoma and ovarian clear cell carcinoma samples was calculated, and the top 100 most variable genes were selected and visualised across normal, endometrioid, and clear cell samples using a heatmap generated with the pheatmap (v1.0.13) package in R.

### 2.6. Uniform Manifold Approximation and Projection Analysis (UMAP)

Cancer samples were subsetted from the combined expression matrix. Gene variance was calculated across cancer samples, and the 2000 most variable genes were selected for dimensionality reduction. Three-dimensional UMAP was performed using the umap R package (v0.2.10.0) and visualised using the plotly (v4.12.0) package for three-dimensional plots, with 15 nearest neighbours, a minimum distance of 0.1, the Euclidean distance metric, and a random seed of 123. The same UMAP embedding was visualised by histotype and dataset to assess whether sample separation reflected histotype or dataset of origin.

### 2.7. Functional Enrichment Analysis

Functional enrichment analysis was performed using the gprofiler2 (v0.2.4) package in R. The correction_method = “fdr” parameter was utilised, with an adjusted *p*-value threshold of 0.05. The background gene universe was defined implicitly as all annotated human genes within the Ensembl database. Enriched terms were ranked by their FDR-adjusted *p*-values. The top-ranked Gene Ontology and Kyoto Encyclopedia of Genes and Genomes pathway terms were visualised using dot plots generated with ggplot2 (v 4.0.2). Genes contributing to each enriched pathway are provided in the Supplementary Tables (Supplementary Tables S17–S20).

### 2.8. Functional mTOR Pathway Visualisation

Gene annotation was performed using the biomaRt package (v2.62.1) in R, querying the Ensembl database (release 115). Ensembl gene identifiers were mapped to their corresponding gene symbols using the hsapiens_gene_ensembl dataset. The KEGGREST (v1.46.0) R package was used to retrieve the list of genes associated with the mTOR signalling pathway (hsa04150) from the Kyoto Encyclopedia of Genes and Genomes (KEGG) database (release 117.0) [13,14]. mTOR DEGs gene symbols were identified and mapped onto the KEGG mTOR signalling pathway using the Pathview (v1.46.0) R package [15].

### 2.9. Prediction and Integration of miRNA–mRNA Regulatory Interactions

Predicted targets of significantly differentially expressed miRNAs (DEMs) were obtained using the multiMiR package (v1.28.0) in R [16]. Genes belonging to the KEGG mTOR signalling pathway (hsa04150) were retrieved using the KEGGREST package (v1.46.0) and intersected with the significantly differentially expressed EAOC genes. Predicted miRNA–target pairs involving these genes were retained only when the interaction was computationally predicted by at least two of the four selected target-prediction databases available through multiMiR, including DIANA-microT, miRanda, miRDB and TargetScan [17,18,19]. Experimentally supported miRNA–target interactions were subsequently retrieved from the multiMiR validated-interaction table using the multiMiR package (v1.28.0) in R, which integrates mainly the TarBase and miRTarBase databases [20,21]. Those overlapping with the predicted candidate miRNA–mTOR pairs were retained, and pairs exhibiting opposing expression patterns, an upregulated miRNA with a downregulated target gene or a downregulated miRNA with an upregulated target gene, were considered putative regulatory pairs and were used to construct the regulatory network. (Supplementary Table S21).

### 2.10. Network Construction and Topological Analysis of mTOR Pathway miRNA–mRNA Interactions

A network analysis of candidate miRNA–mRNA interactions was performed using Cytoscape software (version 3.10.3), developed by the Cytoscape Consortium (Cytoscape Consortium, San Diego, CA, USA) [22]. Hub genes and miRNAs were identified using unweighted degree centrality, defined as the number of regulatory edges incident to a node. A descriptive heuristic threshold of degree centrality > 3 was utilised as an exploratory cutoff to identify highly interconnected nodes as candidate hubs. (Supplementary Table S22).

## 3. Results

### 3.1. Transcriptomic Dysregulation of mTOR Pathway in Endometriosis-Associated Ovarian Cancers (EAOCs)

We initially evaluated six Gene Expression Omnibus (GEO) datasets—GSE230956, GSE189553, GSE226870, GSE121103, GSE295399, and GSE160692—comprising a total of 139 samples for potential inclusion in the analysis. Following preliminary sample-level processing and quality control, 131 samples from these six datasets were retained for cross-study quality assessment. A preliminary leave-one-dataset-out (LODO) sensitivity analysis was then conducted. Although differential expression estimates were largely robust to the exclusion of any single dataset, exclusion of GSE160692 produced the largest change relative to the full six-dataset analysis, with a Spearman correlation of 0.987 for log2 fold changes and 94.8% directional concordance. This indicated that GSE160692 exerted a greater influence on the pooled results than the other datasets. Its distinct separation in principal component analysis (PCA) and atypical expression-density distribution after normalisation further suggested persistent dataset-specific effects. Consequently, GSE160692 was excluded from subsequent analyses. (Supplementary Material File S7, Supplementary Table S10).

The final cancer cohort comprised 120 tumour specimens aggregated from five GEO datasets, including 68 ovarian clear cell carcinoma (OCCC) and 52 endometrioid ovarian carcinoma (EnOC) samples. The comparator cohort consisted of 149 normal ovarian tissue samples obtained from the Genotype-Tissue Expression (GTEx) project.

Differential-expression analysis was performed on 30,862 genes and identified 15,030 genes that were significantly differentially expressed between the EAOCs and normal ovarian tissue (adjusted *p* < 0.05 and absolute log2 fold change > 1), comprising 7432 upregulated and 7598 downregulated genes. A repeated leave-one-dataset-out (LODO) sensitivity analysis across the five retained Gene Expression Omnibus (GEO) datasets demonstrated that Spearman correlation coefficients for log2 fold changes ranged from 0.995 to 1.000, while concordance in the direction of effects ranged from 97.3% to 99.3%. Collectively, these results indicate a high degree of robustness and reproducibility of the differential-expression findings, with no single GEO dataset exerting a disproportionate influence on the overall results. (Supplementary Material File S7, Supplementary Tables S8 and S9).

As a sensitivity analysis, we conducted a random-effects meta-analysis in which differential gene expression was estimated separately for each of the five Gene Expression Omnibus (GEO) cancer datasets relative to the common Genotype-Tissue Expression (GTEx) normal controls, incorporating statistical adjustment for the dependence induced by the shared control group. Among the 30,862 genes evaluated, 16,377 satisfied the significance thresholds of an adjusted *p*-value < 0.05 and an absolute log2 fold change > 1, comprising 9765 upregulated and 6612 downregulated genes. (Supplementary Table S11).

**Figure 2:**
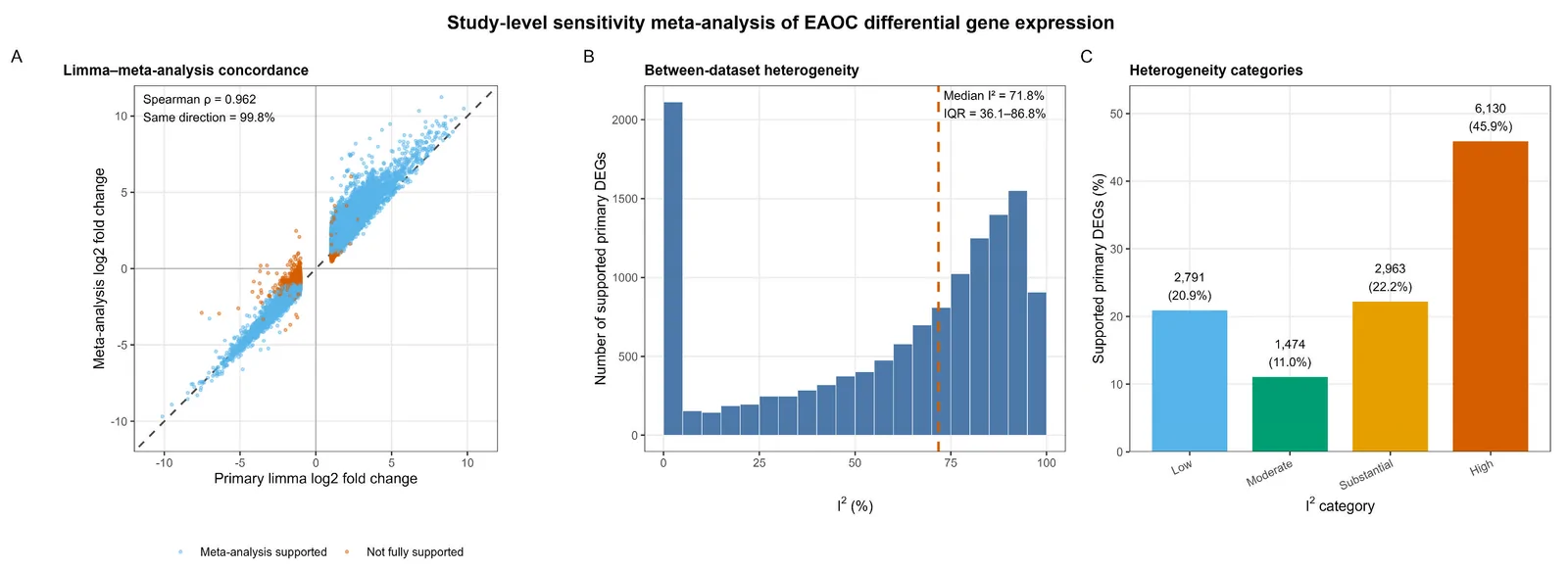
Concordance and between-dataset heterogeneity in the study-level sensitivity meta-analysis in pooled EAOCs versus normal ovarian tissue. (**A**) Concordance between the log2 fold-change estimates from the primary LIMMA analysis and the pooled log2-scale mean expression differences from the study-level random-effects meta-analysis. Each point represents one of the 15,030 LIMMA-identified significantly differentially expressed genes. Blue points represent genes meeting all meta-analytic support criteria, Benjamini–Hochberg-adjusted *p*-value < 0.05, absolute pooled effect > 1 and a pooled effect direction consistent with the LIMMA result, whereas orange points represent genes not meeting all three criteria. The diagonal dashed line represents equal effect estimates between the two analyses. Spearman’s *p* was 0.96, and 99.8% of genes showed the same pooled effect direction. (**B**) Distribution of I^2^ values among the 13,358 LIMMA DEGs that met all meta-analytic support criteria. The orange dashed line represents the median I^2^ of 71.8%; the interquartile range was 36.1–86.8%. (**C**) Classification of heterogeneity among the supported genes as low (I^2^ < 25%), moderate (25% ≤ I^2^ < 50%), substantial (50% ≤ I^2^ < 75%) or high (I^2^ ≥ 75%). The meta-analysis preserved the effect estimates from each of the five GEO cancer datasets and accounted for the covariance introduced by using the same 149 GTEx normal ovarian samples in all five comparisons.

Effect sizes derived from the meta-analysis exhibited a strong correlation with those obtained from the primary pooled differential expression analysis across all 30,862 evaluated genes (Spearman’s *p* = 0.963), with 90.8% demonstrating concordant directions of regulation. Of the 15,030 genes identified as significantly differentially expressed in the primary pooled analysis, 15,027 were represented in the meta-analysis. Within this subset, 13,358 (88.9%) were supported by the sensitivity meta-analysis in terms of statistical significance, direction of regulation, and effect size, while a further 945 (6.3%) retained statistical significance and directional concordance. An additional 687 genes (4.6%) showed the same direction of regulation but did not reach statistical significance. Furthermore, 13,524 primary DEGs showed consistent directions of regulation across all five GEO datasets (Figure 2; Supplementary Table S12).

Despite the strong directional concordance, the magnitude of the expression effects varied between datasets. Among the 13,358 supported primary DEGs, the median I^2^ was 71.8% (interquartile range, 36.1–86.8%). Heterogeneity was classified as low for 2791 genes (20.9%), moderate for 1474 (11.0%), substantial for 2963 (22.2%), and high for 6130 (45.9%). Thus, the sensitivity analysis supported the direction and statistical significance of most findings from the primary analysis, yet the estimated effect sizes differed across datasets (Figure 2).

**Figure 3:**
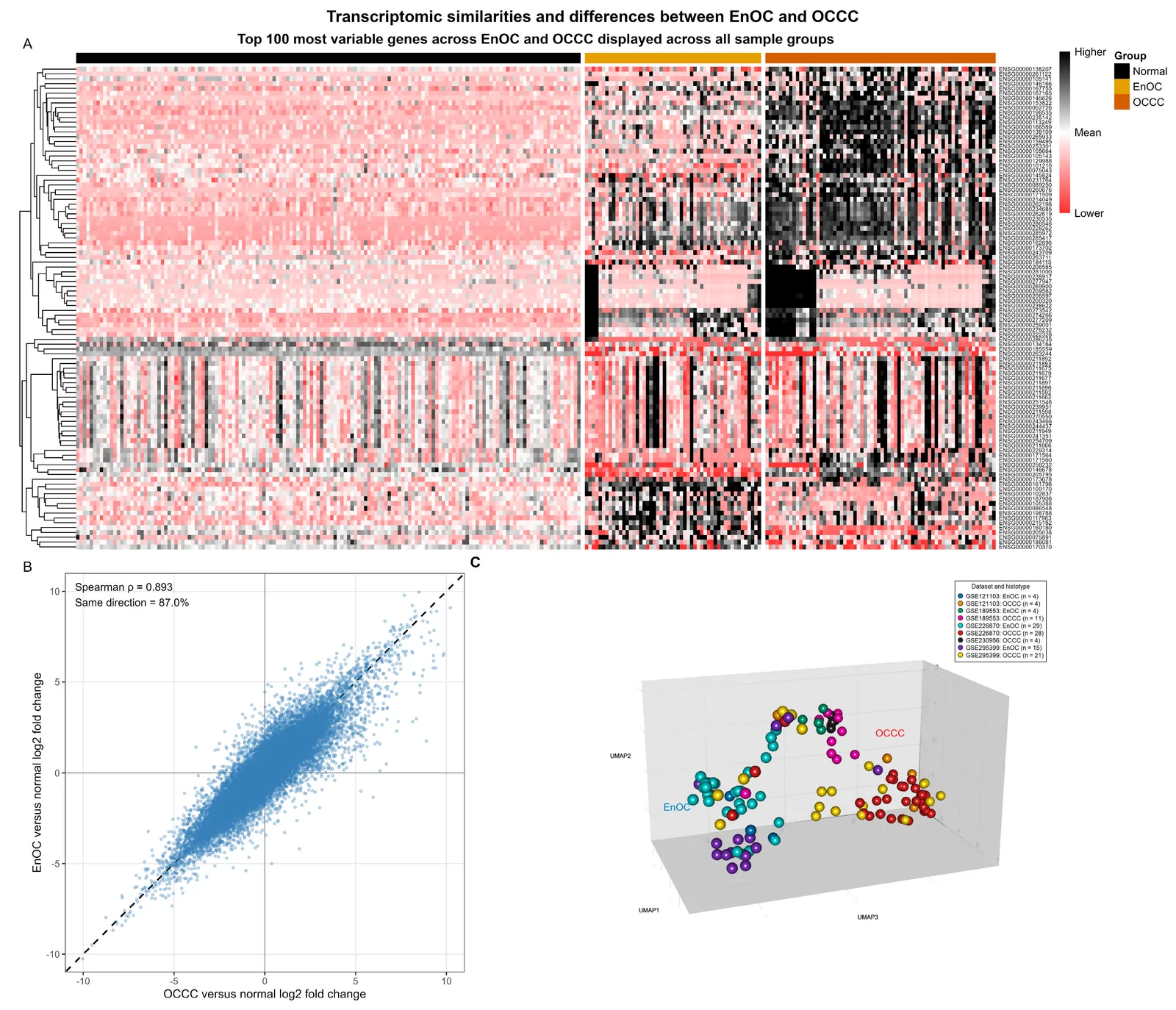
Shared and distinct transcriptomic patterns in ovarian clear cell and endometrioid carcinomas. (**A**) Heatmap showing the 100 genes with the highest expression variance across endometrioid ovarian carcinoma (EnOC) and ovarian clear cell carcinoma (OCCC) samples, displayed across normal ovarian tissue, EnOC and OCCC samples. Expression values were standardised by gene, with red indicating lower expression and black indicating higher expression. Samples were ordered by group without column clustering, while genes were hierarchically clustered using Euclidean distance. (**B**) Concordance of log2 fold changes among 30,866 genes assessed in both ovarian clear cell carcinoma (OCCC) and endometrioid ovarian carcinoma (EnOC). Each point represents the log2 fold changes calculated relative to normal ovarian tissue. Expression changes were strongly correlated between histotypes (Spearman’s *p* = 0.893), and 87.0% of genes showed the same direction of regulation. The dashed diagonal line indicates identical log2 fold changes in both histotypes. (**C**) Three-dimensional uniform manifold approximation and projection (UMAP) of the 120 cancer samples based on the 2000 most variable genes, showing EnOC (*n* = 52) and OCCC (*n* = 68) samples. Each sphere represents an individual tumour sample, coloured according to its dataset of origin and histotype. Samples show partial separation by histotype, with contributions from multiple datasets within each group.

Transcriptional similarity between endometrioid ovarian carcinoma (EnOC) and ovarian clear cell carcinoma (OCCC) was assessed by comparing differential expression profiles relative to normal ovarian tissue. Across the 30,866 genes evaluated in both histotypes, expression changes were strongly correlated (Spearman’s *p* = 0.893), with 87.0% of genes showing concordant directions of regulation, irrespective of reaching the significance thresholds (Figure 3B). Of these, 18,348 were uniquely differentially expressed across both histotypes, and 11,953 (65.1%) were shared, including 5784 upregulated and 6125 downregulated in both. These shared genes demonstrated strong effect-size concordance (Spearman’s *p* = 0.926), with 99.6% regulated in the same direction. A further 6395 genes reached statistical significance in only one histotype, comprising 3439 OCCC-specific and 2956 EnOC-specific genes. Nevertheless, 89.8% of these showed concordant directions of regulation across both histotypes, although the magnitudes of their expression changes were less strongly correlated (Spearman’s *p* = 0.515). Collectively, these findings indicate a shared transcriptional programme consistent with the classification of EnOC and OCCC as endometriosis-associated ovarian cancers (Supplementary Material File S7, Figure S7 and Table S3).

To further investigate these subtype-specific differences, the top 100 most variable genes were selected and visualised in a heatmap across normal, EnOC, and OCCC samples (Figure 3A). Despite the strong effect size concordance observed, the heatmap revealed distinct transcriptomic profiles of each subtype. The Uniform Manifold Approximation and Projection (UMAP) analysis further emphasised that EnOC and OCCC form distinct transcriptional clusters (Figure 3C). These findings indicate that shared transcriptional changes relative to normal tissue coexist alongside histotype-associated differences in gene-expression patterns.

**Figure 4:**
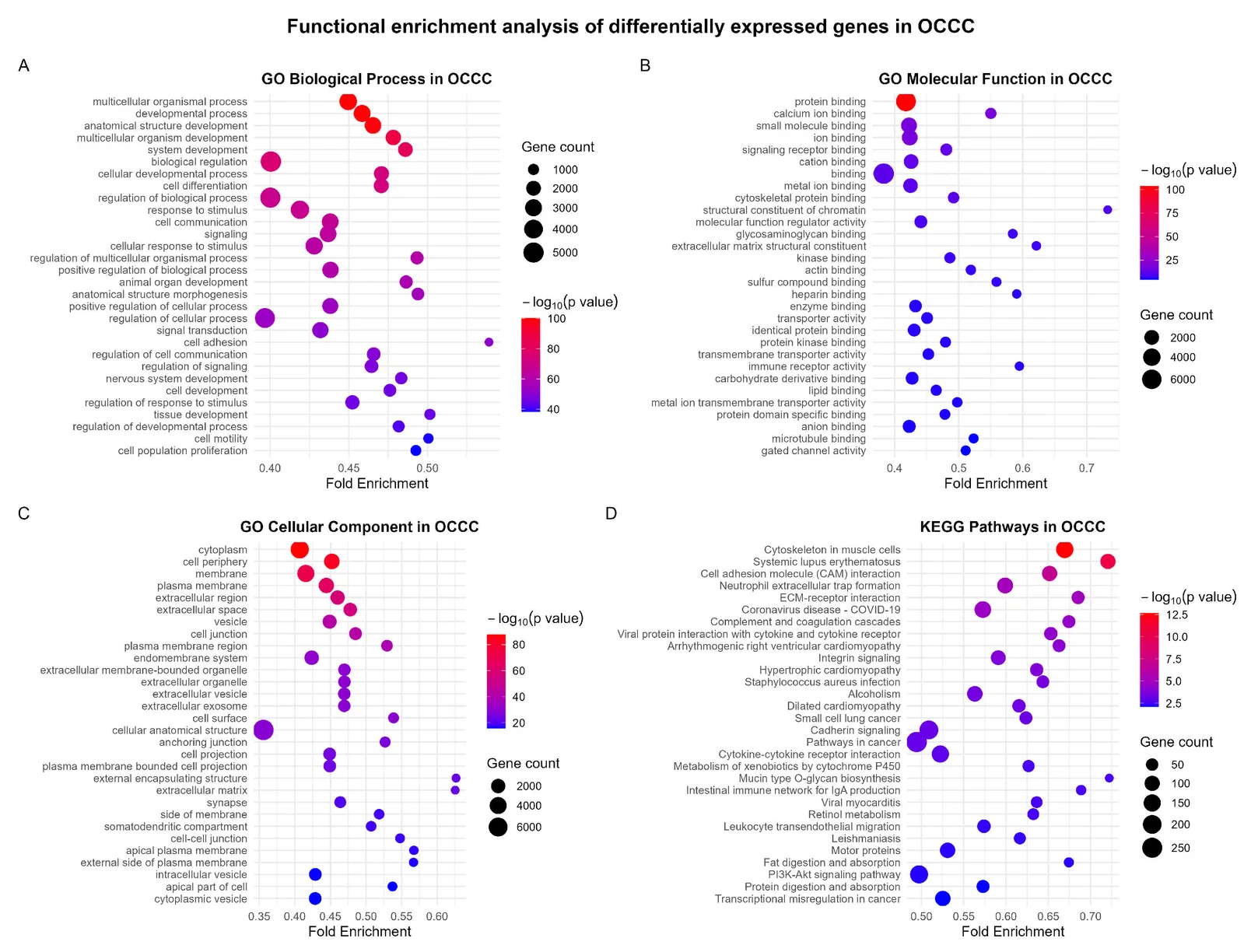
Dot plots showing functional enrichment analysis of differentially expressed genes in OCCC compared with normal ovarian tissue. Results include gene ontology biological process, molecular function, cellular component, and KEGG pathways. The colour gradient reflects statistical significance (−log10 *p*-value). (**A**) Gene Ontology (GO) biological process terms; (**B**) GO molecular function terms; (**C**) GO cellular component terms; (**D**) Kyoto Encyclopedia of Genes and Genomes (KEGG) pathways.

Subsequently, functional enrichment analysis was conducted for each subtype independently to identify significantly enriched biological pathways. Among the top-ranked biological processes in OCCC, cellular response to stimulus (FDR = 3.86 × 10^−63^) and signal transduction (FDR = 1.58 × 10^−51^) contained 57 and 53 mTOR-associated genes, respectively, with WNT7A, WNT7B, TNF, RPS6KA1, and DDIT4 among the leading contributors. Cell population proliferation was also significantly enriched (FDR = 4.42 × 10^−39^) and included 30 mTOR-associated genes, notably WNT7A, WNT7B, TNF, RPS6KA1, IRS1, and AKT3. At the molecular-function level, protein binding (FDR = 1.84 × 10^−104^), ion binding (FDR = 7.36 × 10^−19^), and kinase activity (FDR = 6.81 × 10^−4^) contained 60, 23, and 14 mTOR-associated genes, respectively. The kinase-activity category included RPS6KA1, AKT3, PRKCB, ULK1, PIK3CB, and mTOR. Cellular-component analysis predominantly localised these genes to the cytoplasm, membrane, and cell periphery. KEGG analysis identified enrichment of pathways in cancer (FDR = 8.01 × 10^−4^), containing 28 mTOR-associated genes, including WNT7A, WNT7B, and AKT3. The PI3K–Akt signalling was also enriched (FDR = 5.98 × 10^−3^) and contained 14 mTOR-associated genes, including DDIT4, IRS1, AKT3, PIK3CB, mTOR, PIK3CD, and STK11 (Figure 4) (Supplementary Tables S17 and S18).

**Figure 5:**
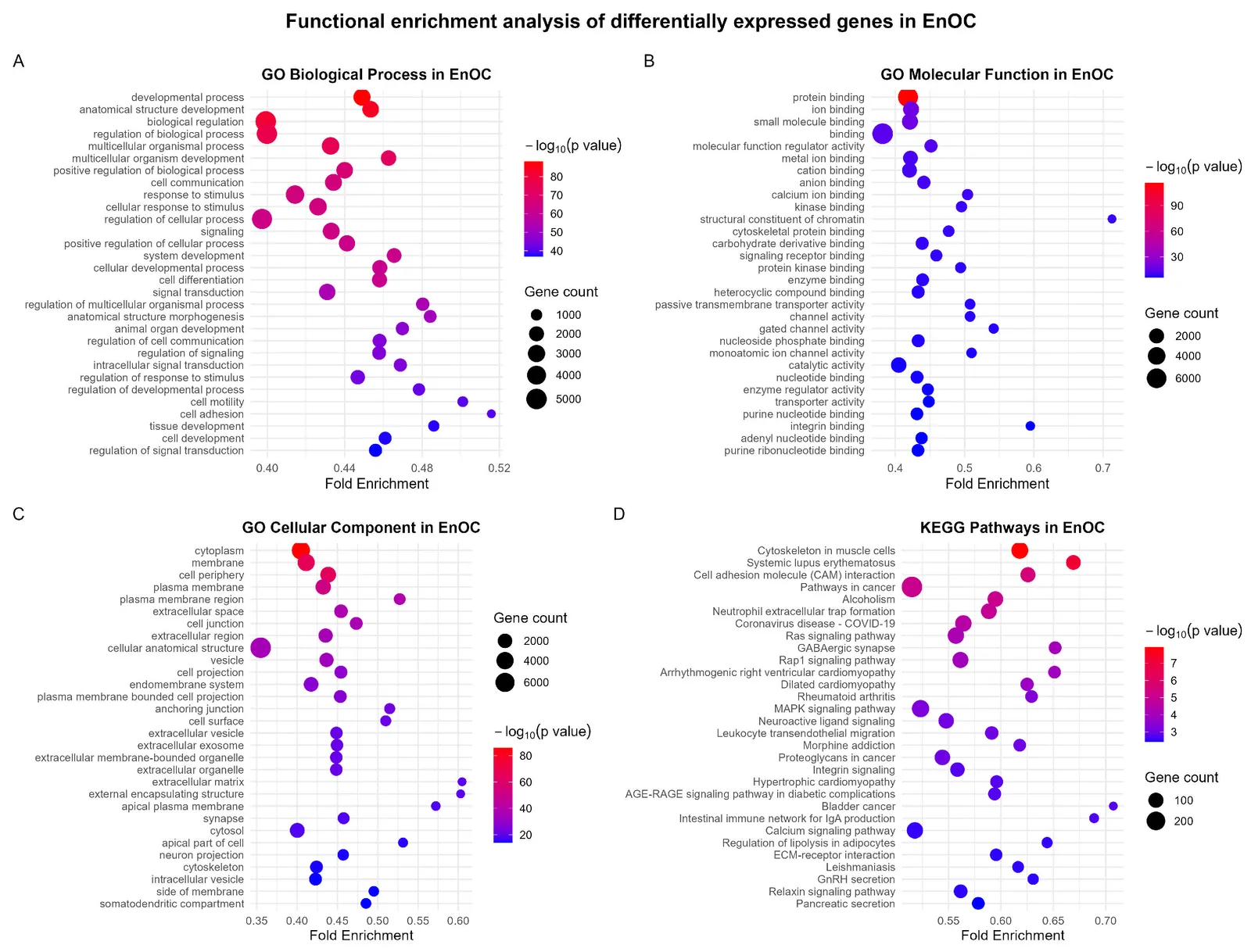
Functional enrichment analysis of DEGs in EnOC compared with normal ovarian tissue. Results include gene ontology biological process, molecular function, cellular component, and KEGG pathways. Colour intensity reflects statistical significance, represented as −log10 (*p*-value). (**A**) Gene Ontology (GO) biological process terms; (**B**) GO molecular function terms; (**C**) GO cellular component terms; (**D**) Kyoto Encyclopedia of Genes and Genomes (KEGG) pathways.

Among the enriched biological processes in endometrioid ovarian carcinoma (EnOC), regulation of cell communication (FDR = 3.81 × 10^−46^), cellular response to stimulus (FDR = 1.86 × 10^−65^), and developmental processes (FDR = 7.33 × 10^−89^) contained 52, 67, and 49 mTOR-associated genes, respectively, with WNT7A, WNT7B, WNT4, DDIT4, RPS6KA1, and AKT3 among the leading contributors. At the molecular-function level, protein binding (FDR = 1.75 × 10^−117^), ion binding (FDR = 2.03 × 10^−19^), and transporter activity (FDR = 1.41 × 10^−6^) contained 70, 33, and five mTOR-associated genes, respectively. The ion-binding category included RPS6KA1, AKT3, SGK1, ULK1, and PIK3CD, whereas transporter activity involved ATP6V1B1, ATP6V1G2, ATP6V1A, ATP6V1C2, and ATP6V1E1. KEGG analysis identified enrichment of cAMP signalling (FDR = 1.30 × 10^−2^), containing eight mTOR-associated genes, including AKT3, PIK3R3, PIK3CD, AKT2, MAPK3, and PIK3CB, and MAPK signalling (FDR = 5.18 × 10^−4^), containing 16 mTOR-associated genes, including RPS6KA1, AKT3, RPS6KA6, TNF, NRAS, and MAPK3 (Figure 5) (Supplementary Tables S19 and S20).

**Figure 6:**
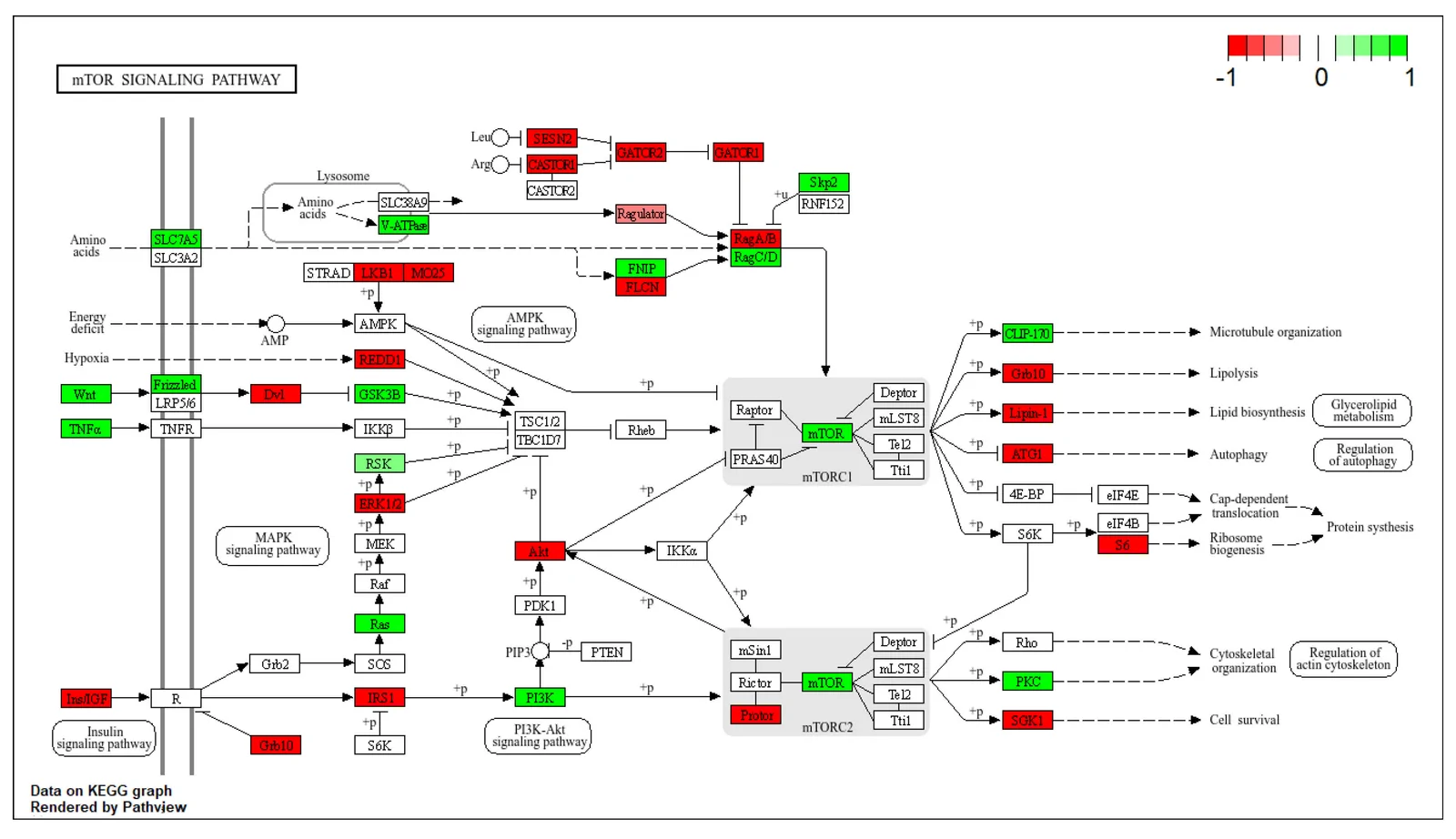
Pathway-level dysregulation of mTOR signalling in OCCC. The diagram overlays differentially expressed genes from the OCCC study cohort onto the KEGG mTOR signalling pathway (hsa04150). Colours indicate the log2 fold change (green for upregulation, red for downregulation). Significance is defined by an FDR-adjusted *p*-value < 0.05. Visualised interactions represent transcriptomic dysregulation and do not establish functional protein activation.

**Figure 7:**
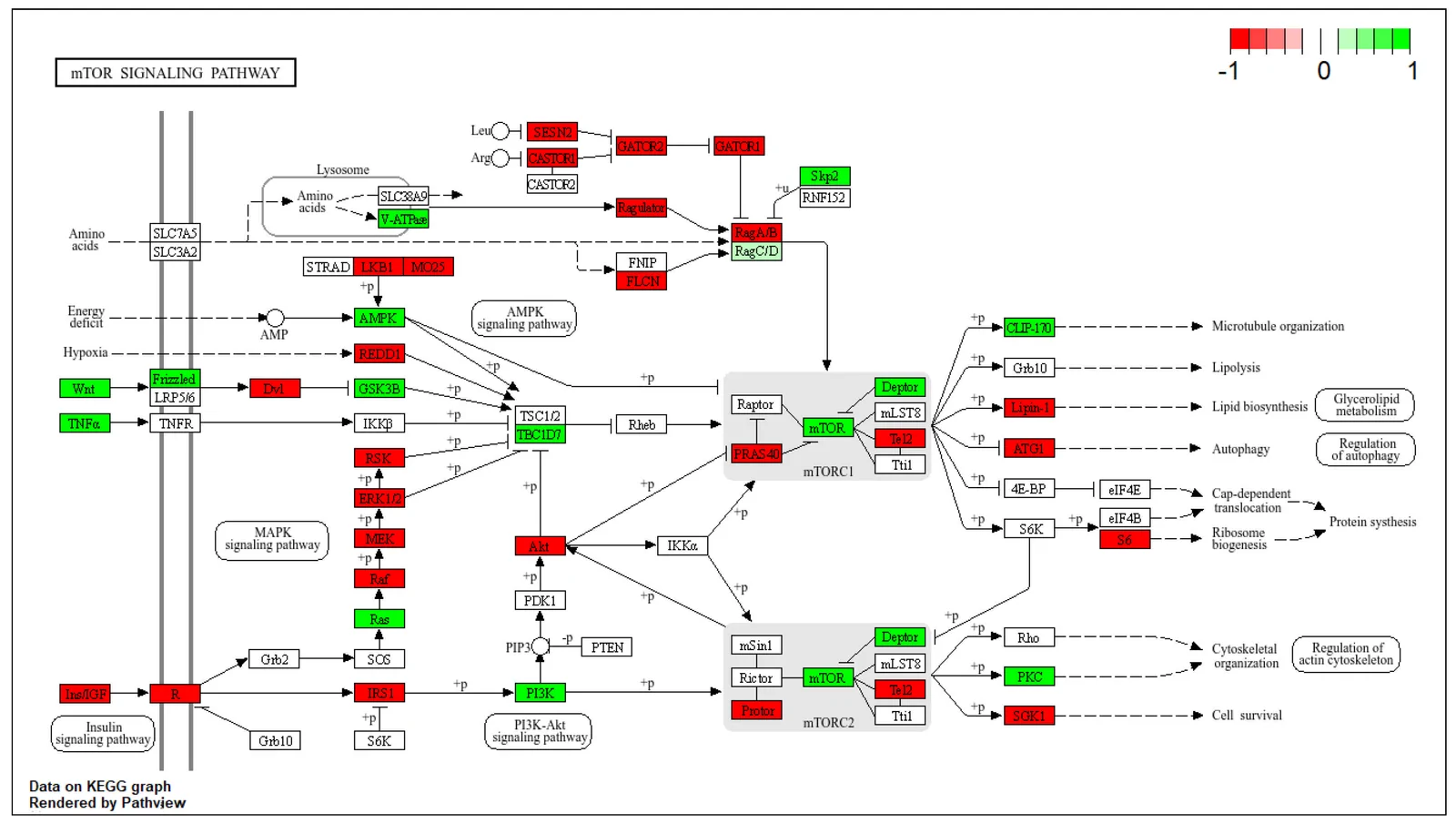
Pathway-level dysregulation of mTOR signalling in EnOC. The diagram overlays differentially expressed genes from the EnOC study cohort onto the KEGG mTOR signalling pathway (hsa04150). Colours indicate the log2 fold change (green for upregulation, red for downregulation). Significance is defined by an FDR-adjusted *p*-value < 0.05. Visualised interactions represent transcriptomic dysregulation and do not establish functional protein activation.

To investigate mTOR pathway dysregulation in each subtype, we overlaid the mTOR DEGs in OCCC and EnOC onto the KEGG mTOR pathway (hsa04150) (Figure 6 and Figure 7).

The mTOR signalling pathway in both subtypes showed upregulation of PIK3CB (encoding the p110β catalytic subunit of class IA PI3K) and mTOR, which suggests transcriptomic dysregulation consistent with potential activation of the canonical PI3K/AKT/mTOR axis in EAOCs [23,24]. Contrary to our previous findings of RICTOR downregulation in high-grade serous ovarian cancers, there was no transcriptional downregulation of RICTOR detected in the EAOCs [5,25]. The observed upregulation of SLC7A5 in OCCC suggested a transcriptomic profile associated with altered amino acid uptake. Although SLC7A5 did not reach statistical significance in the EnOC differential expression analysis, it showed a similar direction of regulation, indicating possible pathway rewiring and the absolute need for functional validation.

**Figure 8:**
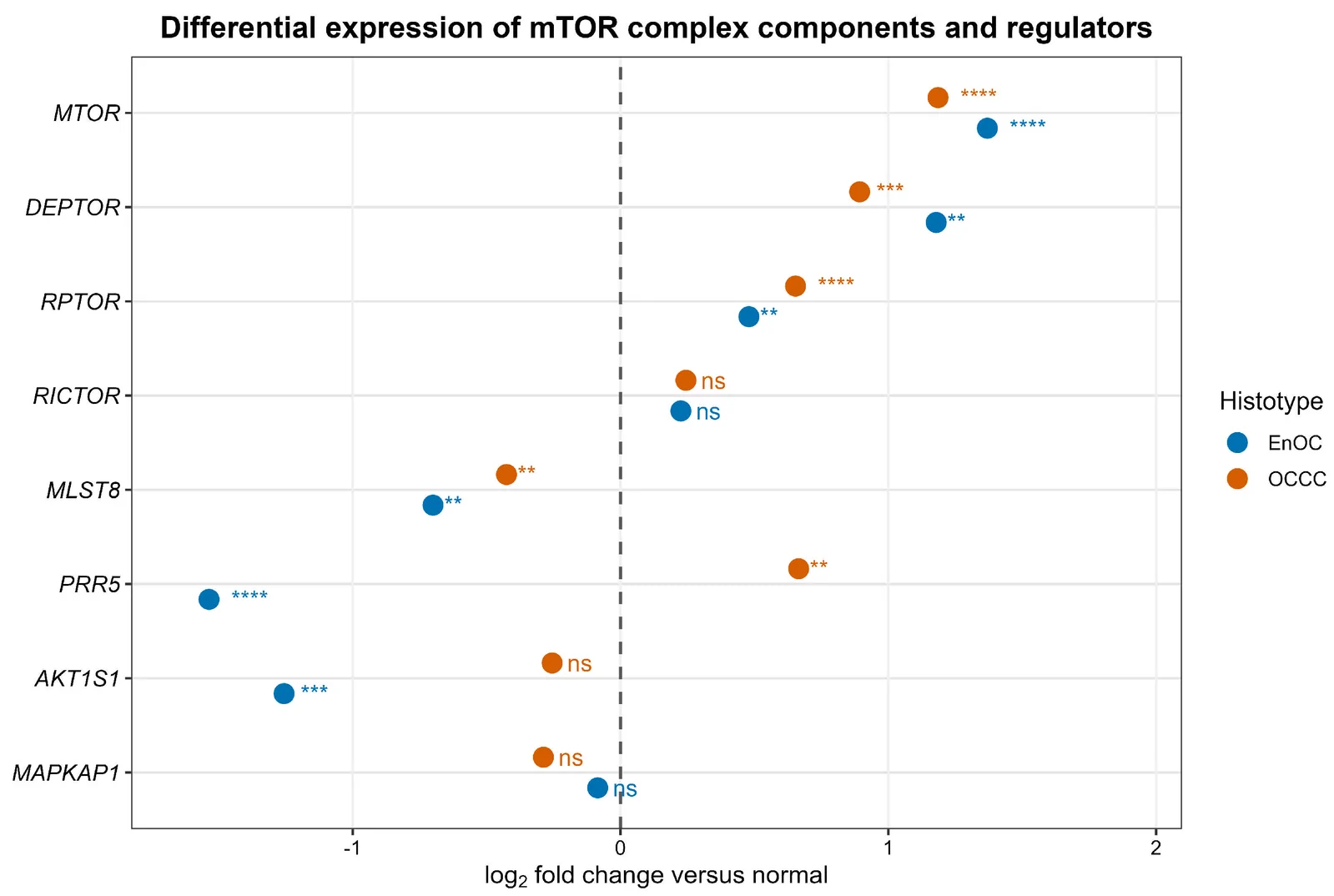
Differential expression of mTOR complex components and associated regulators in ovarian clear cell and endometrioid carcinomas. Forest plot showing log2 fold changes for selected mTOR complex genes in endometrioid ovarian carcinoma (EnOC; blue) and ovarian clear cell carcinoma (OCCC; orange), each compared with normal ovarian tissue. Points represent estimated log2 fold changes. The dashed vertical line represents no change in expression. Asterisks indicate the statistical significance of each histotype-versus-normal comparison based on adjusted *p*-values: ** < 0.01, *** < 0.001, and **** < 0.0001; ns, not significant. No direct comparison between EnOC and OCCC is represented.

To further investigate histotype-specific transcriptomic alterations in the mTOR signalling pathway, we compared the expression levels of core mTOR complex-encoding genes in each ovarian carcinoma histotype relative to normal ovarian tissue. (Figure 8).

mTOR, DEPTOR, and RPTOR were significantly upregulated in both OCCC and EnOC, whereas MLST8 was significantly downregulated in both, demonstrating concordance in both direction and statistical significance. RICTOR showed a similar direction of change in both histotypes but did not reach statistical significance in either. AKT1S1 and MAPKAP1 were directionally concordant, with reduced expression in both histotypes. However, AKT1S1 reached statistical significance only in EnOC, while MAPKAP1 was not significant in either. In contrast, PRR5 exhibited statistically significant changes in opposite directions, with increased expression in OCCC and decreased expression in EnOC. Collectively, these findings indicate that broader mTOR pathway dysregulation is accompanied by predominantly concordant transcriptomic changes in selected mTOR complex genes, alongside histotype-associated differences in specific components.

### 3.2. The Regulatory miRNA Network of mTOR Pathway in Endometriosis-Associated Ovarian Cancers (EAOCs)

Five miRNA expression datasets were included in the analysis: four from the Gene Expression Omnibus (GSE200852, GSE230956, GSE134289, and GSE261800) and one from the European Genome-phenome Archive (EGAD00001009636). Collectively, these datasets comprised 82 endometrioid ovarian carcinoma (EnOC) samples, 55 ovarian clear cell carcinoma (OCCC) samples, and 33 normal ovarian tissue samples. The number of miRNAs detected per dataset ranged from 761 to 2137. In total, 2652 miRNAs were detected across all datasets, of which 650 passed low-abundance filtering criteria and were retained for downstream analyses. (Supplementary Table S13).

Differential expression analysis identified 188 significantly differentially expressed miRNAs (DEMs) between the EAOCs and normal ovarian tissue (adjusted *p* < 0.05 and absolute log2 fold change > 1), of which 131 were upregulated and 57 were downregulated.

**Figure 9:**
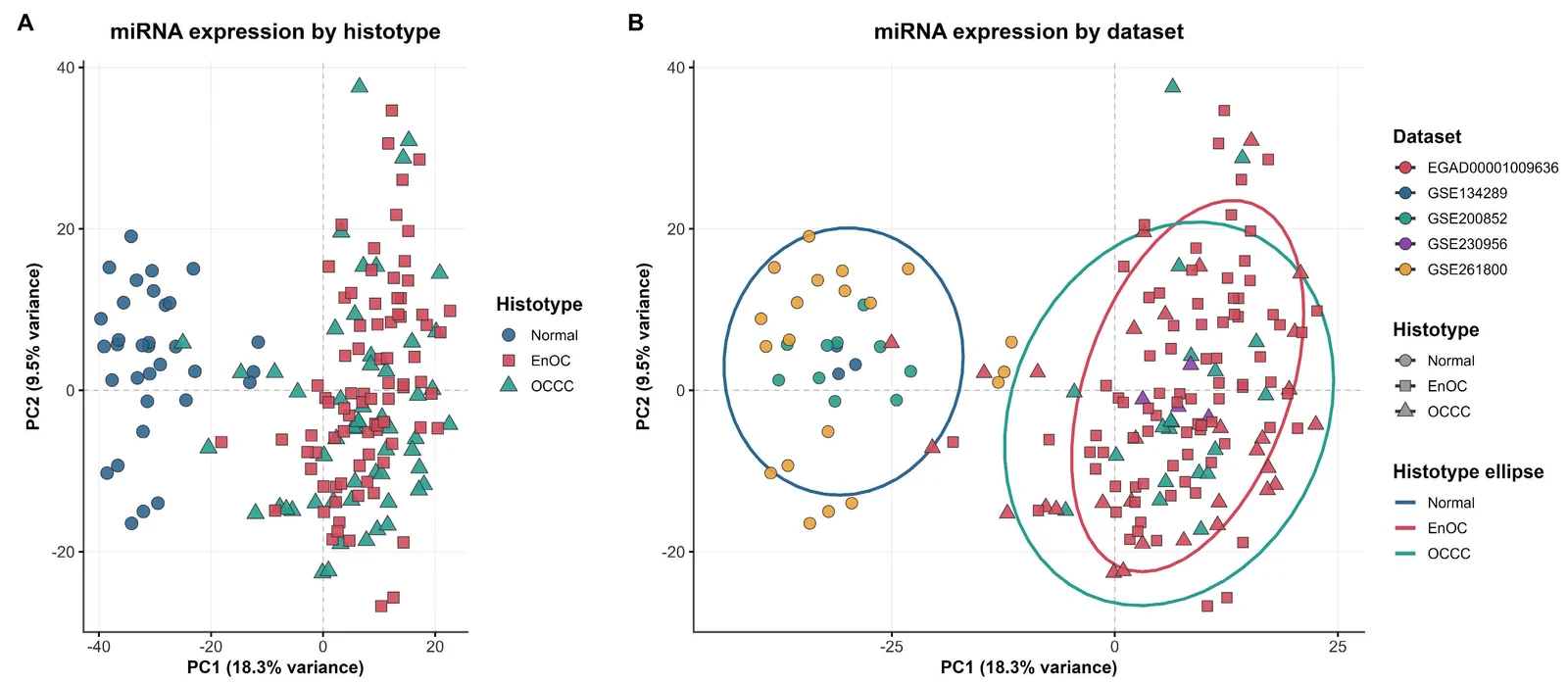
Principal component analysis of batch-corrected microRNA expression stratified by histotype and dataset. Principal component analysis was conducted on the 500 most variable micRNAs following variance-stabilising transformation and correction for dataset-specific batch effects. (**A**) Samples are colour- and shape-coded according to histotype: normal ovarian tissue, endometrioid ovarian carcinoma (EnOC), and ovarian clear cell carcinoma (OCCC). (**B**) Samples are colour-coded by dataset of origin and shape-coded by histotype. Ellipses denote 80% confidence contours for each histotype. The first and second principal components account for 18.3% and 9.5% of the total variance, respectively.

A principal component analysis (PCA) of the variance-stabilised miRNA expression is provided in the Supplementary Material (Supplementary Material File S7, Figure S8). Following batch-effect correction, normal ovarian tissue samples segregated from the ovarian carcinoma samples, whereas endometrioid ovarian carcinoma and ovarian clear cell carcinoma samples exhibited substantial overlap in the PCA space. However, because all endometrioid ovarian carcinoma samples were derived exclusively from the EGA dataset, putative histotype-specific differences could not be reliably disentangled from dataset-specific (batch) effects (Figure 9).

Leave-one-dataset-out analysis showed strong concordance across four iteration models (Spearman’s *p* = 0.997–1.000; directional concordance = 95.8–99.7%). However, exclusion of GSE200852 resulted in a rank-deficient model because disease status became completely confounded with the dataset. GSE200852 was the only cohort containing both malignant and normal samples and therefore provided the principal within-dataset information for estimating the cancer–normal effect after batch adjustment. Accordingly, the pooled miRNA findings are considered exploratory, and therefore, subsequent analyses were interpreted at the pooled EAOC level rather than as evidence of histotype-specific miRNA regulation (Supplementary Table S14).

Candidate target genes of the significantly differentially expressed miRNAs (DEMs) in the study cohort were identified using a two-step approach. Initially, four computational databases accessible through the multiMiR package in R, DIANA-microT, miRanda, miRDB, and TargetScan, were queried [16,17,18,19]. Their prediction algorithms employ complementary yet partially distinct features, such as seed-sequence complementarity, duplex thermodynamic stability, evolutionary conservation, target-site accessibility, and local sequence context. To increase the likelihood of identifying potential biologically relevant regulatory pairs, only interactions predicted by at least two databases were retained [17,26]. Considering the complex many-to-many relationships between miRNAs and mRNAs, candidate pairs were further refined by retaining only those with some degree of experimental support. The experimentally supported databases integrated within the multiMiR package include interactions identified through high-throughput methods such as HITS-CLIP, PAR-CLIP, and CLASH, as well as low-throughput methods such as Luciferase reporter assays and GFP reporter assays, which have been manually curated from published studies [20,26]. The term “validated” does not necessarily indicate direct, pair-specific functional validation, and the type and strength of evidence may vary among the records. Furthermore, validation experiments were not necessarily performed in ovarian cancers or EAOCs and included unrelated tissues. Experimentally supported interactions that overlapped with the predicted candidate pairs were retained, and those exhibiting inverse expression patterns were considered potentially putative regulatory interactions. Although such patterns are consistent with the canonical repressive effects of miRNAs, inverse expression alone does not establish direct binding or causal relationships and would require confirmation through targeted functional experiments [26,27].

**Figure 10:**
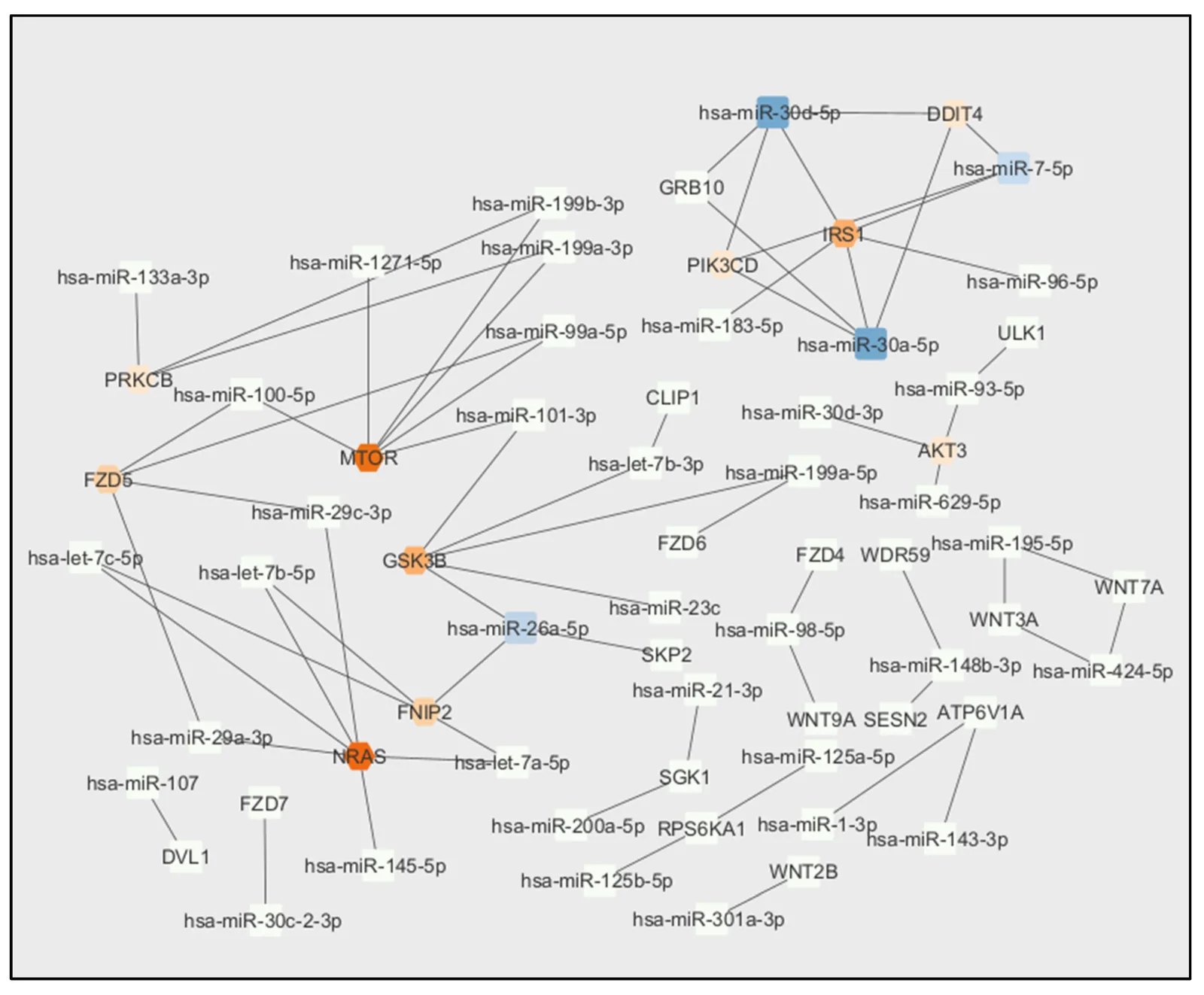
miRNA–mRNA interaction network involving mTOR pathway-associated genes in endometriosis-associated ovarian cancers (EAOCs). The network depicts inverse relationships between differentially expressed miRNAs and putative mTOR pathway-related target genes in EAOCs. Blue nodes indicate candidate hub miRNAs, orange nodes indicate candidate hub target genes, and white nodes denote additional miRNAs and genes included in the network. Candidate hubs were identified using an exploratory degree-centrality threshold of >3 connections. Because miRNA and mRNA expression profiles were derived from non-matched samples, the network represents putative regulatory associations rather than direct, sample-level evidence of regulatory interactions within the analysed cohort.

A total of 65 putative regulatory miRNA–mRNA interaction pairs were identified, encompassing 27 differentially expressed mTOR pathway genes (DEGs) and 38 differentially expressed miRNAs (DEMs) in EAOCs, and an interaction network was subsequently constructed (Figure 10). (Supplementary Table S21).

Degree centrality was calculated to describe node connectivity within the network, and nodes with three or more connections were highlighted for exploratory visualisation. Among the miRNAs, hsa-miR-30a-5p, hsa-miR-30d-5p, hsa-miR-26a-5p and hsa-miR-7-5p showed the highest connectivity. Among the mTOR-pathway genes, mTOR and NRAS had the highest degree centrality, followed by GSK3B, IRS1, FNIP2, FZD5, PRKCB, DDIT4, PIK3CD and AKT3. These findings describe the topological connectivity of nodes within the predicted network and should not be interpreted as evidence of causality (Table 3).

**Figure 11:**
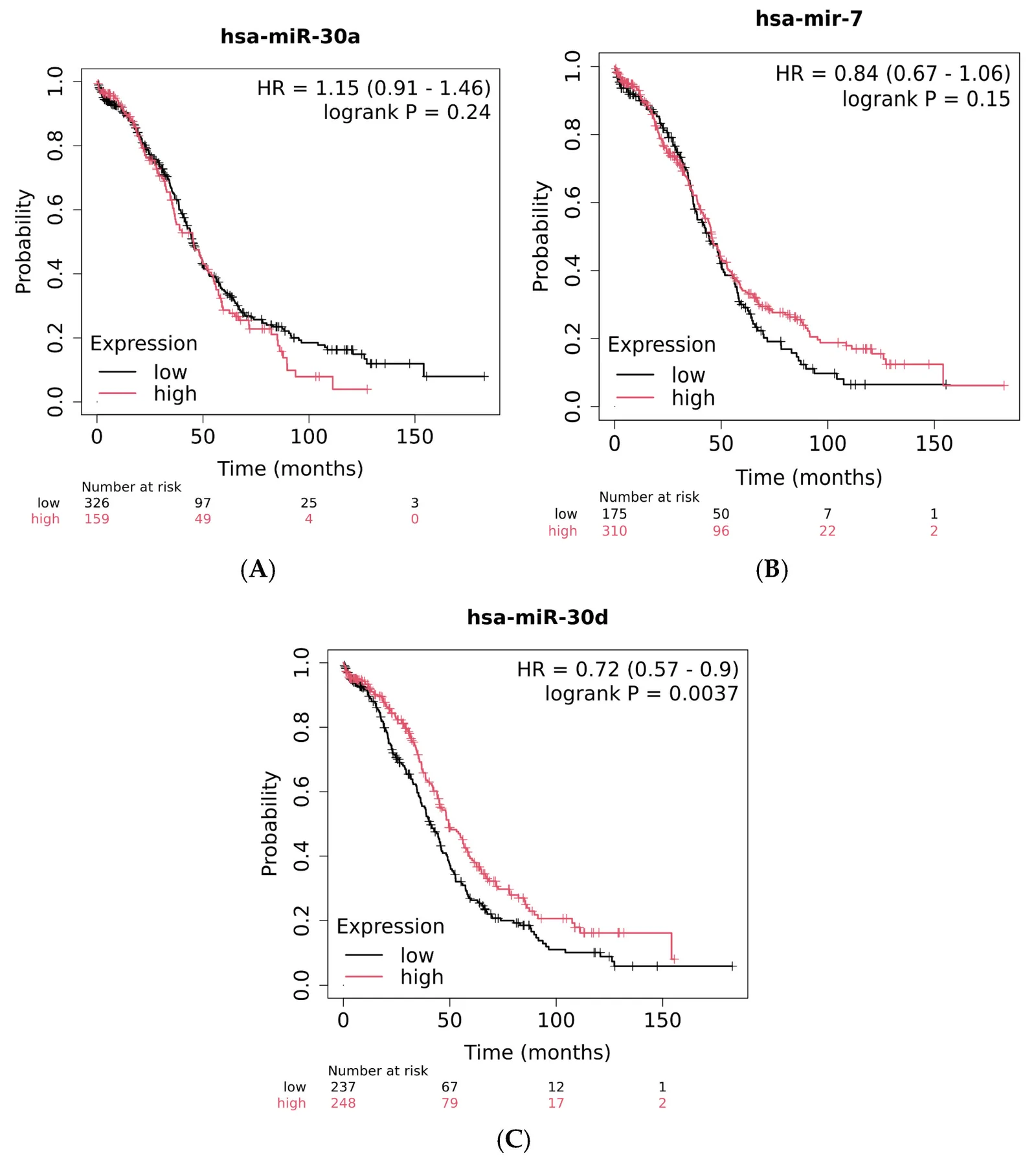
Kaplan–Meier survival analysis stratified by miRNA expression. Kaplan–Meier curves depict survival probabilities in patients with low (black) versus high (red) expression of (**A**) hsa-miR-30a, (**B**) hsa-miR-7, and (**C**) hsa-miR-30d. Hazard ratios (HRs) with corresponding 95% confidence intervals (CIs) and log-rank *p*-values are reported within each panel. Tick marks denote censored observations, and the number of patients at risk at each time point is shown below the curves. Elevated hsa-miR-30d expression was significantly associated with improved overall survival (HR = 0.72, 95% CI: 0.57–0.90; log-rank *p* = 0.0037), whereas expression levels of hsa-miR-30a and hsa-miR-7 were not significantly associated with survival outcomes. These survival curves were generated using the Pan-cancer miRNA KM Plotter (https://kmplot.com/analysis/index.php?p=service&cancer=pancancer_mirna). The primary limitation of this analysis is the inability to distinguish between high-grade serous ovarian carcinoma (HGSOC) and endometriosis-associated ovarian carcinomas (EAOCs).

Due to the lack of publicly available follow-up data, we were unable to perform survival analysis (Kaplan–Meier), specifically for hsa-miR-30a-5p, hsa-miR-30d-5p, and hsa-miR-7-5p. However, we were able to obtain data for hsa-miR-30a, hsa-miR-30d, and hsa-miR-7 precursor transcripts, which are not expected to exhibit substantial functional differences relative to their corresponding mature forms. Of the three miRNAs, only hsa-miR-30d demonstrated prognostic value when we used the Pan-cancer miRNA KM Plotter (https://kmplot.com/analysis/index.php?p=service&cancer=pancancer_mirna, accessed on 18 August 2026). The only limitation of this analysis is that we are unable to differentiate between HGSOC and EAOCs (Figure 11).

## 4. Discussion

PI3K/AKT/mTOR dysregulation is frequently reported in endometriosis-associated ovarian cancers and is often attributed to genomic aberrations, particularly involving PIK3CA, PTEN, and ARID1A genes [28,29]. In this study, our integrative in silico analysis provides a transcriptomic and miRNA-based characterisation of the mechanistic target of rapamycin (mTOR) signalling in endometriosis-associated ovarian cancers (EAOCs), highlighting both shared and subtype-specific potential regulatory features across the two histotypes.

We demonstrated distinct transcriptional signatures of ovarian clear cell carcinoma (OCCC) and endometrioid ovarian carcinoma (EnOC) in line with the previously published literature [30,31,32]. Despite transcriptomic differences between the subtypes, we observed upregulation of PIK3CB and mTOR in both OCCC and EnOC, with preserved expression of RICTOR. Collectively, these findings may indicate transcriptomic rewiring of the canonical mTOR signalling pathway in both subtypes, with conservation of mTORC2 component expression in EAOCs [25]. These findings are consistent with previous studies using the MDAH-2774 ovarian cancer cell line [33,34].

The functional enrichment analysis revealed a significant overrepresentation of the PI3K–Akt signalling pathway in OCCC, whereas the MAPK/cAMP signalling pathway was preferentially enriched in EnOC. These observations indicate that the upstream, parallel pathways contributing to transcriptomic mTOR signalling dysregulation may differ between histological subtypes and exhibit subtype-specific characteristics [35,36].

MicroRNAs are well-known post-transcriptional regulators of the PI3K/AKT/mTOR signalling network that can simultaneously modulate different components of the pathway to ensure a sustained oncogenic signalling cascade [37,38]. We observed substantial overlap in miRNA expression profiles between ovarian clear cell carcinoma (OCCC) and endometrioid ovarian carcinoma (EnOC) in our study, making investigating putative subtype-specific differences and similarities beyond the scope of the present study. miRNA dysregulation was evaluated in aggregate across endometriosis-associated ovarian cancers (EAOCs) [39,40,41].

Differential expression analysis of the miRNA regulatory network identified several significantly upregulated putative hub miRNAs, including hsa-miR-30a-5p, hsa-miR-30d-5p, hsa-miR-7-5p, and hsa-miR-26a-5p. Downregulation of miRNA-30a has been reported under conditions of oxidative stress, a process known to play a pivotal role in endometriosis-associated carcinogenesis. In the present study, the predicted target genes of hsa-miR-30a-5p, DDIT4 and PIK3CD, are components of the PI3K/AKT/mTOR signalling pathway, whose dysregulation may contribute to tumorigenesis [42,43].

We further demonstrated upregulation of hsa-miR-30d-5p in EAOCs relative to normal ovarian tissue, in agreement with the previous findings by Lee et al. Interestingly, their study showed that elevated expression of miR-181d, miR-30c, miR-30d, and miR-30e-3p was associated with significantly improved disease-free and overall survival outcomes [44].

We observed upregulation of hsa-miR-7-5p in the pooled endometriosis-associated ovarian cancers (EAOCs) cohort. However, the expression and functional role of hsa-miR-7-5p have not been well characterised specifically in ovarian clear cell carcinoma (OCCC) or endometrioid ovarian carcinoma (EnOC). Therefore, its potential relevance to these histotypes remains uncertain. The available ovarian cancer evidence is derived mainly from serous ovarian carcinoma, which is biologically distinct from OCCC and EnOC. In serous ovarian carcinomas, Swiercz et al. reported higher miR-7 expression in TP53-mutated tumours and an association between increasing miR-7 expression and tumour grade among TP53-wild-type tumours, although miR-7 expression was not associated with disease-free survival [45].

Evidence from other tumour types further indicates that the biological role of miR-7 is context-dependent. In lung cancer models, miR-7 exhibited potential oncogenic effects downstream of the EGFR–Ras–ERK–Myc pathway through targeting ERF. Conversely, experimental studies in glioblastoma and tongue squamous cell carcinoma reported potential tumour-suppressive effects through inhibition of EGFR/AKT signalling and targeting of IGF1R, respectively. Although these studies demonstrate that miR-7 can influence cancer-related signalling pathways, they do not establish its function or target relationships in OCCC or EnOC. Consequently, the observed upregulation of hsa-miR-7-5p in the present study should be regarded as a candidate association requiring histotype-specific functional validation [46,47,48].

Beyond mTOR-specific targets, our network identified major highly connected predicted target genes, including GSK3B (Wnt signalling) and NRAS (MAPK signalling) in the miRNA regulatory network. These findings indicate that most of the aforementioned miRNAs are unlikely to act in isolation. Rather, they appear to converge on and co-ordinately modulate multiple oncogenic signalling pathways that are aberrantly regulated in the tumour phenotype [49].

Although GRB10 did not satisfy the descriptive degree-centrality threshold defined in our study, it should be noted that this cutoff was introduced primarily as a heuristic to aid in the identification of highly connected genes and is, by design, largely descriptive rather than inferential. Thus, the simultaneous downregulation of IRS1, GRB10, DDIT4, and PIK3CD, all identified as putative targets within the same miRNA regulatory framework, hsa-miR-30a-5p, hsa-miR-30d-5p, and hsa-miR-7-5p, points to a potentially coordinated transcriptional reprogramming of the PI3K/AKT/mTOR signalling network. These genes include both upstream activators (IRS1, PIK3CD) and regulatory inhibitors or feedback components (GRB10, DDIT4). In particular, downregulation of DDIT4 may relieve inhibitory pressure on mTORC1, whereas reduced IRS1 and PIK3CD expression may reflect diminished reliance on receptor-mediated signalling. Collectively, these findings suggest rewiring of the PI3K/AKT/mTOR axis with potentially increased baseline transcriptomic dysregulation in these histotypes [50,51,52].

Our findings may be interpreted more informatively within the recently proposed distinction between endometriosis-correlated ovarian carcinomas (EROCs) and endometriosis-incidental ovarian carcinomas (EIOCs), a framework that shifts emphasis from the mere co-occurrence of endometriosis and ovarian cancer to the biological relationship between the two lesions [36,53]. In a series of 170 patients, Mezzapesa et al. separated carcinomas showing histological transitional elements, namely atypical endometriosis or borderline lesions bridging benign ectopic endometrium and malignancy, from tumours in which benign endometriosis was an incidental finding and from ovarian carcinomas arising without endometriosis [54]. The resulting groups were histologically and clinically distinct. Endometrioid (62.5%) and clear cell (27.1%) carcinomas predominated among endometriosis-correlated cases, whereas high-grade serous carcinoma accounted for most endometriosis-incidental tumours (59%). Because OCCC and EnOC are precisely the histotypes that dominate the endometriosis-correlated category, they may be regarded as prototypical EROCs. Accordingly, molecular features shared by both, the coordinated transcriptomic dysregulation of PI3K/AKT/mTOR signalling and the potential convergent miRNA regulatory architecture described here, are better considered candidate correlates of endometriosis-driven carcinogenesis than incidental resemblances between two unrelated tumour entities.

Two interpretations of this shared signature nevertheless remain tenable. In the first, the upregulation of PIK3CB and mTOR, the concurrent downregulation of IRS1, GRB10, DDIT4 and PIK3CD, and the predicted convergence of hsa-miR-30a-5p, hsa-miR-30d-5p, hsa-miR-7-5p and hsa-miR-26a-5p upon these transcripts represent a programme inherited from the endometriotic epithelium of origin, in which oxidative stress, ARID1A loss and PI3K/AKT activation are demonstrable before frank malignancy and are progressively acquired across the benign–atypical–carcinoma sequence [28,29]. In this study, the predicted miRNA–mTOR axis would constitute a molecular hallmark of the EROC spectrum. In the second, the same phenotype reflects potential transcriptomic convergent selection for mTORC1-dependent growth in two biologically distinct diseases and would therefore be expected in mTOR-driven tumours irrespective of an endometriotic origin. Our data are compatible with both interpretations, since the public bulk transcriptomic datasets analysed here lack annotation of concurrent endometriosis, atypical endometriosis or histological transitional lesions; resolving the question will require EROC/EIOC-stratified cohorts, ideally with paired sampling of carcinoma, contiguous atypical endometriosis and distant benign endometriosis from the same patient, together with comparison against histotype-matched EIOCs. Conversely, the distinctive upstream enrichment we observed, PI3K–Akt signalling in OCCC versus MAPK/cAMP signalling in EnOC, may indicate that a common downstream mTOR output may be reached through different upstream routes and provides a transcriptomic correlate of the biological heterogeneity acknowledged within the EROC category. This distinction carries translational rather than merely taxonomic weight. If the miRNA–mTOR signature is proven to be EROC-defining, it could be developed towards markers of endometriosis-derived origin and, potentially, of malignant risk in atypical endometriosis. If it reflects convergence, its value would lie instead in flagging mTOR-pathway dependency independently of endometriosis status. In either case, the subtype-specific transcriptomic upstream differences suggest that combination strategies accompanying mTOR inhibition may need to be histotype-adapted, with PI3K-directed in OCCC and MAPK-directed in EnOC, even where the downstream node targeted is shared.

A key strength of this study lies in the identification of EAOC-specific candidate regulatory networks and their potential to generate hypotheses for future experimental investigation through integrative analysis of transcriptomic and miRNA data. Combining gene expression and miRNA profiling offers a more comprehensive systems-level understanding of pathway dysregulation. Several limitations of this study should be acknowledged. First, the work is inherently exploratory and computational in nature. All findings are derived from in silico transcriptomic analyses and, therefore, require experimental validation at both the protein and functional levels. The reliance on GTEx as the sole source of normal ovarian tissue for the transcriptomic analysis constitutes an additional limitation, as disease status is confounded with data source, and residual technical differences between cohorts cannot be fully excluded. Both mRNA and miRNA datasets were comparable with respect to assay type. However, library-preparation kits, sequencing platforms, read configurations, and laboratory procedures were not identical across studies and may have been incompletely reported in some cases. Additionally, disparities in age, tissue quality, RNA integrity, tumour purity and cellular composition may have influenced the observed expression patterns. Although all raw data were processed through a unified computational pipeline, this approach cannot fully eliminate pre-analytical or platform-related variability. Residual technical confounding, therefore, remains possible.

To confirm the proposed regulatory network relationships, a series of dedicated experimental studies will be required. For instance, Western blotting and phosphoproteomic analyses (e.g., quantification of p-AKT, p-mTOR, and 4EBP1) could be employed to validate activation of the implicated signalling pathways. Moreover, because the current data are derived from bulk RNA sequencing, the concurrent dysregulation of putative targets represents an averaged transcriptomic signal across heterogeneous cell populations. Future single-cell and spatial transcriptomic approaches will be necessary to determine whether these miRNA regulatory networks operate within individual cells or primarily reflect subclonal and microenvironmental heterogeneity at the tissue level.

In addition, 3′-UTR luciferase reporter assays and functional studies using miRNA mimics or inhibitors (miR-30a-5p, miR-30d-5p, miR-7-5p, miR-26a-5p) will be important to directly assess the interactions between these miRNAs and their target genes. The absence of phosphoproteomic or other direct phosphorylation data currently limits inferences regarding the activation status of the mTOR complexes. Finally, the relatively limited number of EAOC-specific datasets constrains the generalisability of the findings and underscores the need for larger, well-annotated, disease-specific cohorts.

## 5. Conclusions

This study provides an in silico integrated transcriptomic and post-transcriptional overview of the mTOR signalling pathway in EAOCs. We identified hsa-miR-30a-5p, hsa-miR-30d-5p, hsa-miR-7-5p, and hsa-miR-26a-5p as potential central hub nodes within the mTOR signalling network in this gynaecological malignancy. The concurrent downregulation of IRS1, GRB10, DDIT4, and PIK3CD, which were identified as candidate targets of hsa-miR-30a-5p, hsa-miR-30d-5p, and hsa-miR-7-5p, suggests potential coordinated transcriptional reprogramming of the PI3K/AKT/mTOR network. Together with the observed miRNA co-regulation of Wnt, MAPK and mTOR, this suggests potential post-transcriptional modulation of oncogenic pathway crosstalk in EAOCs. This exploratory computational work provides a hypothesis-generating framework for future studies aimed at functional validation, which will be necessary before informing the development of targeted therapeutic strategies that account for both transcriptional and post-transcriptional regulatory mechanisms in these histotypes.

## Figures and Tables

**Table 1:** Characteristics of the GEO datasets included in the mRNA analysis.

Accession No.	Platform	Sample Type	Total Number of Samples	No. of Selected Samples	
				OCCC	EnOC
GSE230956	Illumina NextSeq 500 (Illumina, San Diego, CA, USA)	Ovary	8	4	-----
GSE189553	Illumina HiSeq 2500 (Illumina)	Ovary	23	11	4
GSE226870	Illumina HiSeq 2500 (Illumina)	Ovary	57	28	29
GSE121103	Illumina NextSeq 500 (Illumina)	Ovary	59	4	4
GSE295399	Illumina NovaSeq 6000 (Illumina)	Ovary	147	21	15
Total Selected				68	52

**Table 2:** Characteristics of the miRNA datasets included in the analysis.

Accession No.	Platform	Sample Type	Total Number of Samples	No. of Selected Samples		
				OCCC	EnOC	Normal
GSE200852	Illumina MiSeq (Illumina)	Ovary	52	20	-----	10
GSE230956	Illumina NextSeq 500 (Illumina)	Ovary	16	4	------	--------
GSE134289	HiSeq X Ten (Illumina)	Ovary	6	-------	-------	3
GSE261800	Ion Torrent Proton (Thermo Fisher Scientific, Waltham, MA, USA)	Ovary	40	-------	-------	20
EGAD00001009636	Illumina NextSeq 500 (Illumina)	Ovary	215	31	82	----
Total Selected				55	82	33

**Table 3:** mTOR pathway potential hub nodes in EAOCs.

Node	Degree Centrality	Node Type
NRAS	6	Gene
mTOR	6	Gene
GSK3B	5	Gene
IRS1	5	Gene
FNIP2	4	Gene
FZD5	4	Gene
PRKCB	3	Gene
DDIT4	3	Gene
PIK3CD	3	Gene
AKT3	3	Gene
hsa-miR-30a-5p	4	miRNA
hsa-miR-30d-5p	4	miRNA
hsa-miR-26a-5p	3	miRNA
hsa-miR-7-5p	3	miRNA

## Data Availability

The original contributions presented in this study are included in the article/Supplementary Materials. Further inquiries can be directed to the corresponding author.

## Supplementary Materials

The following supporting information can be downloaded at https://www.mdpi.com/article/10.3390/biomedicines14091991/s1.
