# Supplementary material for: Integrative mRNA and miRNA Profiling Identifies Shared and Subtype-Associated PI3K–AKT–mTOR Pathway Dysregulation and Candidate miRNA-Mediated Regulatory Interactions in Endometriosis-Associated Ovarian Cancers (EAOCs)
Source: Biomedicines. 2026 Sep 4;14(9):1991. doi: 10.3390/biomedicines14091991 (PMC13604469; doi:10.3390/biomedicines14091991)
Supplement: Supplementary file 1 [file biomedicines-14-01991-s001.zip › Supplementary Materials File S7.pdf]

# Sensitivity Analysis and Diagnostic Plots:

## mRNA quality control and diagnostic visualisation

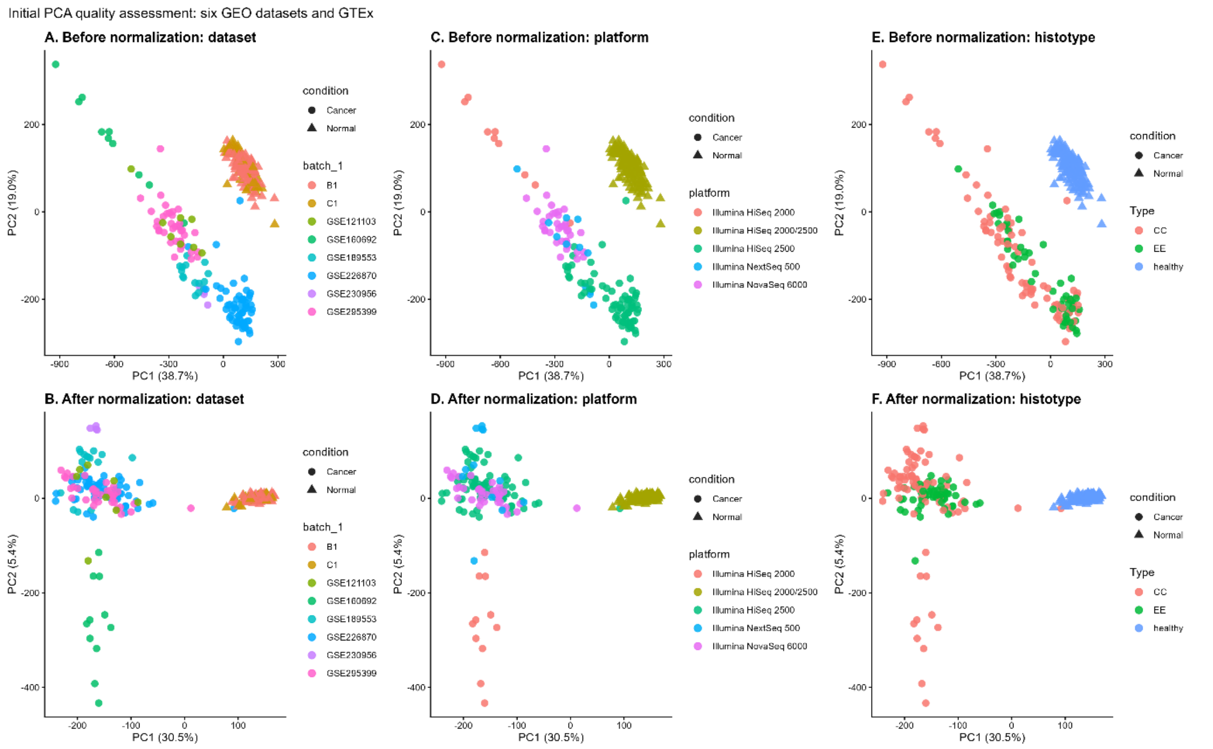


**Figure S1. Principal component analysis of the integrated cohort before and after cross-study normalization.**

Principal component analysis (PCA) of log2-transformed expression data from six GEO tumour datasets (GSE121103, GSE160692, GSE189553, GSE226870, GSE230956 and GSE295399) and GTEx normal tissue samples (batches B1 and C1). Panels A, C and E show the data before normalization, and panels B, D and F show the same samples after normalization. The PCA projections were computed independently; therefore, their axis scales and percentages of explained variance are not directly comparable. Points are coloured by dataset of origin (A, B), sequencing platform (C, D) or histological type (E, F), and shaped by condition (circles, cancer; triangles, normal). Before normalization, PC1 and PC2 explained 38.7% and 19.0% of the total variance, respectively, and samples clustered predominantly according to dataset and sequencing platform. Following normalization, the separation associated with dataset and platform was reduced, with PC1 and PC2 explaining 30.5% and 5.4% of the variance, respectively, although a subset of GSE160692 samples remained displaced towards strongly negative PC2 values. Cancer and normal samples remained separated after normalization; however, because disease condition is confounded with data source, normal samples originated from GTEx, whereas cancer samples originated primarily from GEO datasets, this separation cannot be attributed exclusively to biological differences between cancer and normal tissue. The overall pattern was consistent when samples were coloured by dataset, sequencing platform and histological type. CC, clear cell; EE, endometrioid.

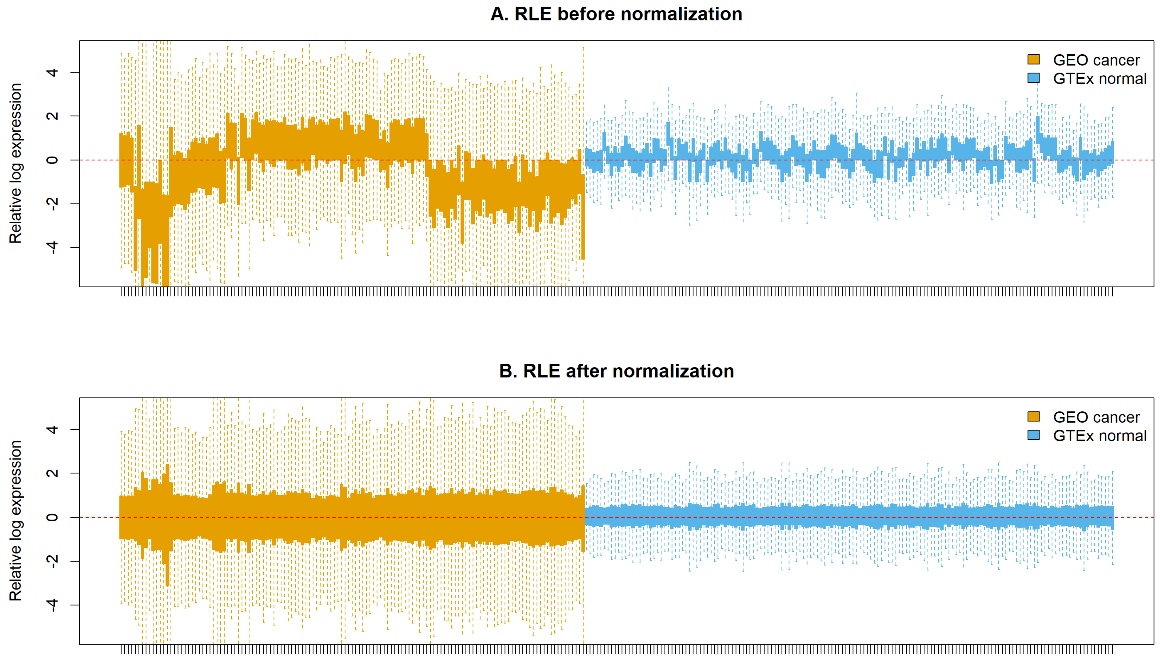


**Figure S2. Relative log expression across samples before and after cross-study normalization.**

Relative log expression (RLE) boxplots for every sample in the integrated cohort, ordered by group: GEO tumour samples (orange) (6 datasets) followed by GTEx normal samples (blue). For each gene, the median expression across all samples was subtracted from the per-sample value; a well-behaved sample therefore shows a median close to zero (red dashed line) and a narrow interquartile range. (A) Before normalization, GEO tumour samples display large, systematically offset medians and highly heterogeneous dispersion, with blocks of samples shifted consistently above or below zero, a signature of study-level technical variation rather than biological signal. (B) After normalization, sample medians are centred on zero, and interquartile ranges are comparable across both the GEO and GTEx samples. Boxes show the interquartile range; whiskers extend to 1.5× the interquartile range. Sample identifiers are omitted from the x-axis for legibility.

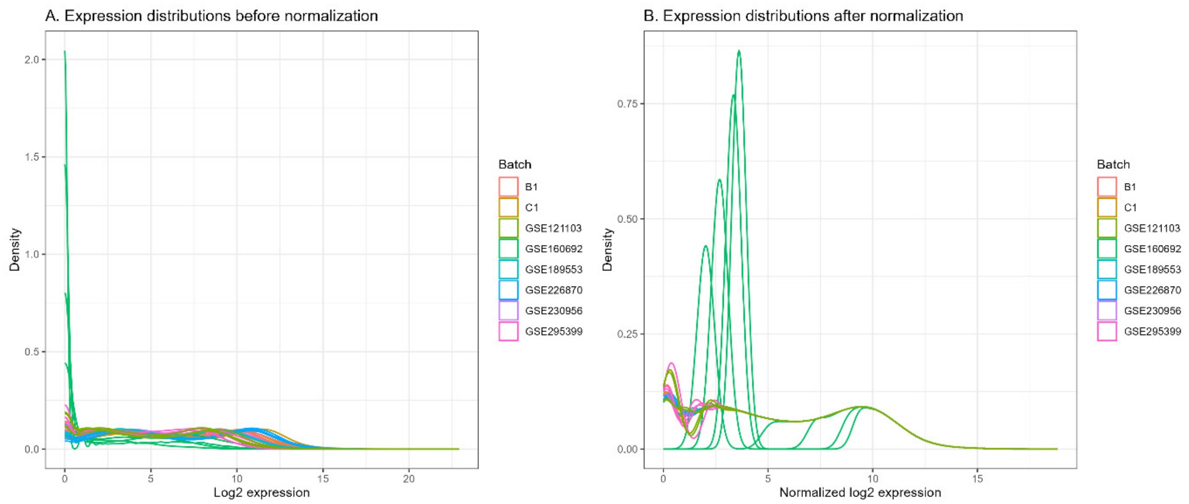


**Figure S3. Per-sample expression density distributions before and after cross-study normalization.**

Kernel density estimates of per-sample expression values, with one curve per sample coloured by dataset of origin. (A) Before normalization, log2 expression distributions differ markedly between studies in both the height of the zero-inflated peak and the position of the expressed-gene mode, with GSE160692 showing an extreme spike at zero. (B) After normalization, the majority of datasets converge onto a common distributional shape, but GSE160692 samples retain sharp, mutually offset modes at low expression values and fail to align with the remainder of the cohort.

***Table S7.1. Leave-one-dataset-out sensitivity analysis across the six original GEO datasets.***

Abbreviations: DEG, differentially expressed gene; LODO, leave-one-dataset-out. Overlap was calculated relative to the significant genes identified in the complete six-dataset analysis.

| Excluded dataset | Excluded samples | Remaining cancer | Remaining normal | Significant DEGs | Overlap with full cohort | Overlap (%) | Spearman correlation | Direction concordance (%) |
|---|---|---|---|---|---|---|---|---|
| GSE121103 | 8 | 123 | 149 | 14,029 | 13,860 | 94.7 | 0.999 | 98.9 |
| GSE160692 | 11 | 120 | 149 | 15,030 | 13,507 | 92.3 | 0.987 | 94.8 |
| GSE189553 | 15 | 116 | 149 | 14,662 | 14,035 | 95.9 | 0.996 | 97.2 |
| GSE226870 | 57 | 74 | 149 | 13,991 | 13,732 | 93.8 | 0.998 | 98.4 |
| GSE230956 | 4 | 127 | 149 | 14,956 | 14,279 | 97.6 | 0.997 | 97.8 |
| GSE295399 | 36 | 95 | 149 | 13,565 | 13,398 | 91.5 | 0.999 | 98.7 |

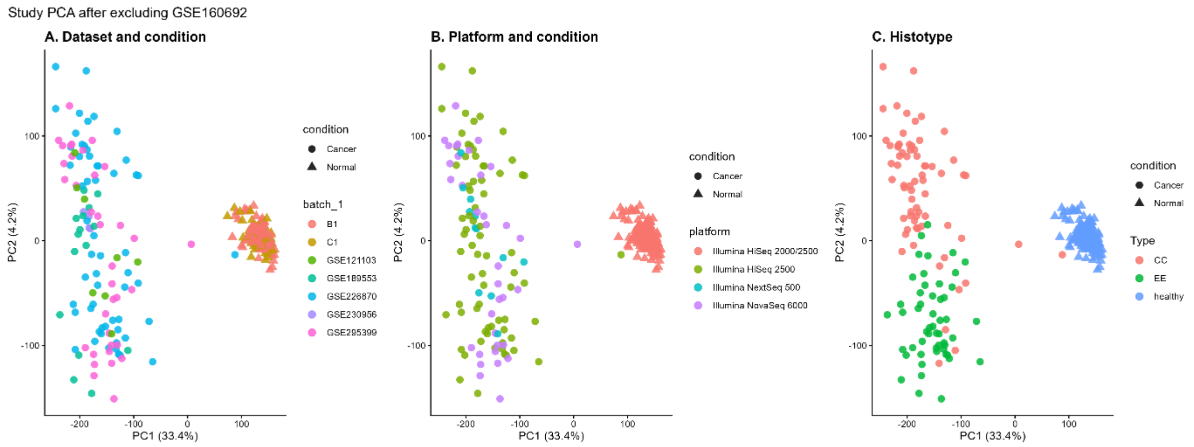


**Figure S4. Principal component analysis of the integrated cohort following exclusion of GSE160692.**

Principal component analysis (PCA) of the normalized, log2-transformed expression profiles from the five retained GEO cancer datasets and GTEx normal ovarian tissue following exclusion of GSE160692. Samples are coloured by dataset of origin (A), sequencing platform (B), or histological type (C), and shaped by disease condition (circles, cancer; triangles, normal). Principal components 1 and 2 explained 33.4% and 4.2% of the total variance, respectively. Cancer samples from the retained GEO datasets showed overlap, although some residual dataset- and platform-associated structure remained. Clear cell (CC) and endometrioid (EE) cancer samples showed partial separation along PC2 but were not completely distinct. Normal GTEx samples formed a separate cluster along PC1; . CC, clear cell; EE, endometrioid.

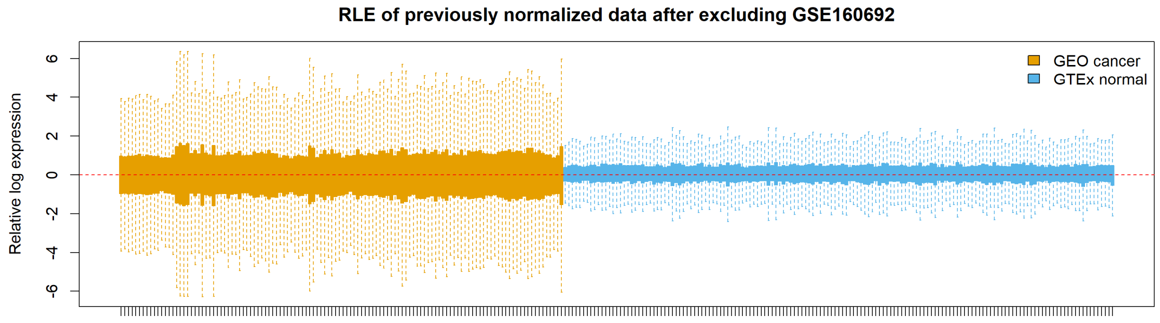


**Figure S5. Relative log expression distributions of the normalized integrated cohort following exclusion of GSE160692.**

Relative log expression (RLE) distributions for the normalized expression profiles of samples from the five retained GEO cancer datasets and GTEx normal ovarian tissue following exclusion of GSE160692. Each boxplot represents the gene-level RLE distribution of one sample; GEO cancer samples are shown in orange and GTEx normal samples in blue. The RLE distributions were consistently centred near zero, indicating comparable sample-wise centring following normalization. The dashed red line denotes an RLE value of zero.

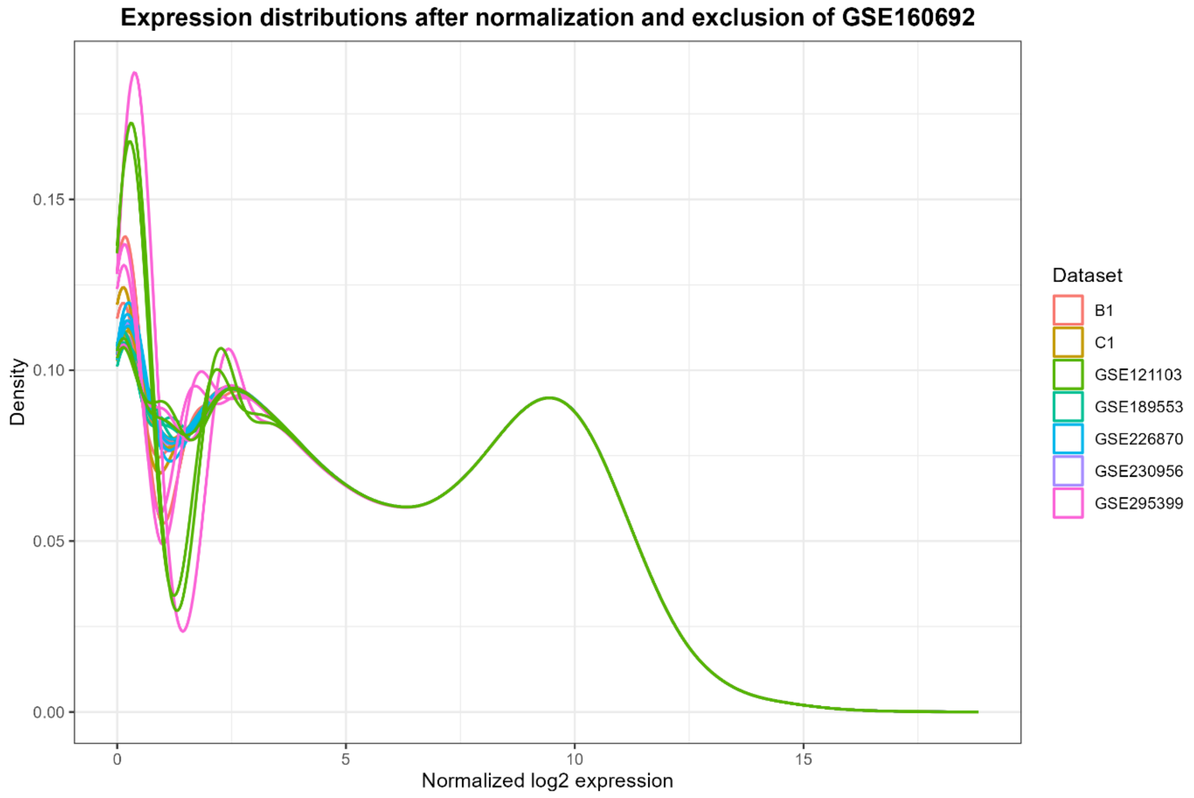


**Figure S6. Expression-density distributions of the normalized integrated cohort following exclusion of GSE160692.**

Kernel-density distributions of normalized log2 expression values for representative samples from the five retained GEO cancer datasets and GTEx normal ovarian tissue batches B1 and C1 following exclusion of GSE160692. Each line representing the gene-expression distribution of one sample and colours indicating dataset or batch of origin. Most samples showed broadly overlapping expression distributions after normalization, indicating improved distributional comparability across studies.

***Table S7.2. Leave-one-dataset-out sensitivity analysis across the five retained GEO datasets.***

| Excluded dataset | Excluded samples | Remaining cancer | Remaining normal | Significant DEGs | Overlap with full five-dataset cohort | Overlap (%) | Spearman correlation | Direction concordance (%) |
|---|---|---|---|---|---|---|---|---|
| GSE121103 | 8 | 112 | 149 | 15,078 | 14,907 | 99.2 | 1.000 | 99.3 |
| GSE189553 | 15 | 105 | 149 | 14,934 | 14,399 | 95.8 | 0.996 | 97.4 |
| GSE226870 | 57 | 63 | 149 | 15,324 | 14,486 | 96.4 | 0.995 | 97.3 |
| GSE230956 | 4 | 116 | 149 | 15,069 | 14,814 | 98.6 | 0.999 | 99.0 |
| GSE295399 | 36 | 84 | 149 | 15,064 | 14,557 | 96.9 | 0.997 | 97.9 |

Abbreviations: DEG, differentially expressed gene; LODO, leave-one-dataset-out□Overlap was calculated relative to significant genes identified in the complete five-dataset primary analysis□

-

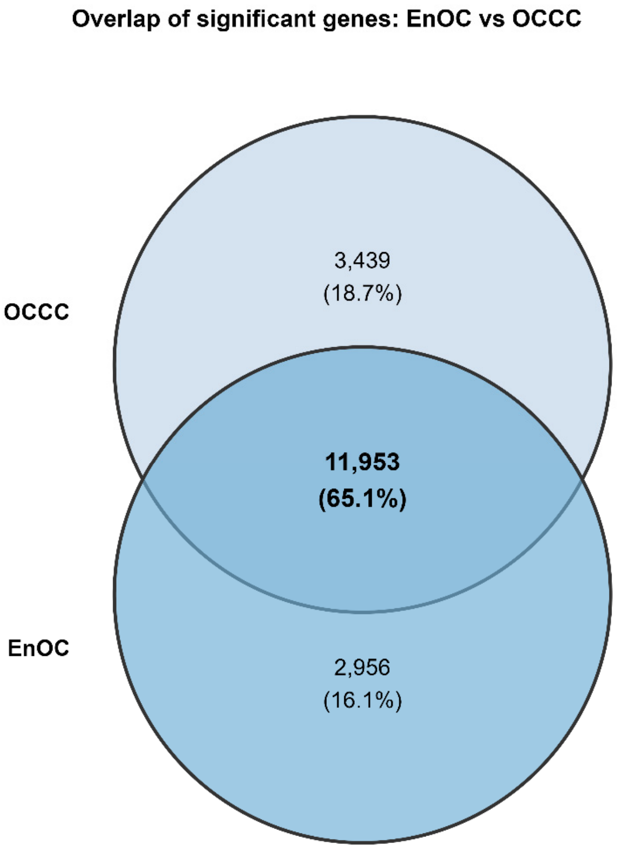


**Figure S7. Overlap of differentially expressed genes between ovarian clear cell and endometrioid ovarian carcinomas.**

Venn diagram showing differentially expressed genes shared between ovarian clear cell carcinoma (OCCC) and endometrioid ovarian carcinoma (EnOC), and those unique to each histotype. Differentially expressed genes were defined by an adjusted P value $< 0.05$ and an absolute $\log_2$ fold change $> 1$. Percentages were calculated using the total number of unique DEGs across both histotypes as the denominator (n = 18,348), where 18,348 = 3,439 OCCC-specific + 11,953 shared + 2,956 EnOC-specific genes. Thus, each percentage was calculated as the number of genes in that Venn-diagram region divided by 18,348 and multiplied by 100.

*Table S7.3. Concordance of differential gene-expression changes between ovarian clear cell carcinoma and endometrioid ovarian carcinoma.*

| analysis | Number of genes | Spearman rho | Same direction percentage |
|---|---|---|---|
| All commonly tested genes | 30866 | 0.893302571 | 87.04399663 |
| Genes significant in both histotypes | 11953 | 0.926063361 | 99.63189158 |
| Genes significant in one histotype only | 6395 | 0.51508112 | 89.80453479 |

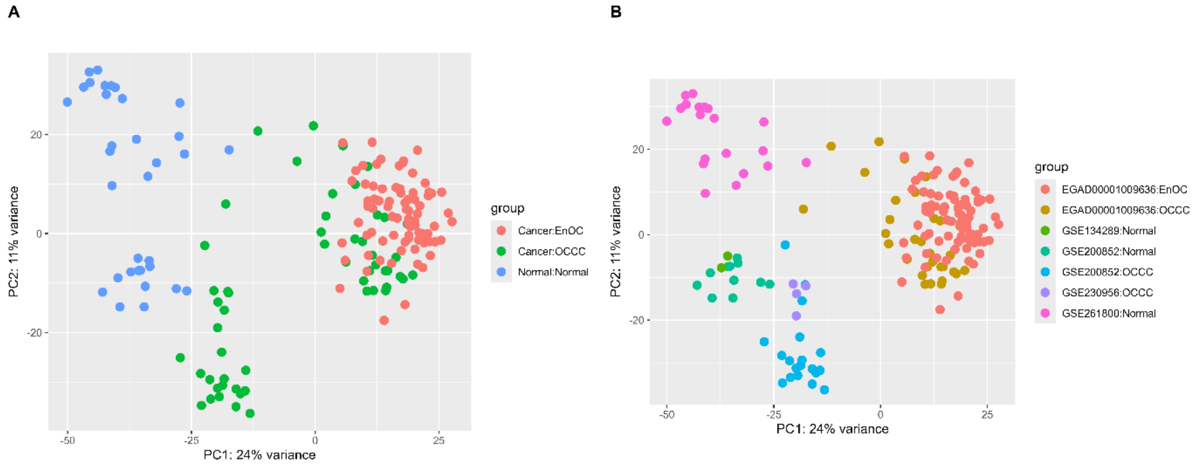


**Figure S8. Principal component analysis of variance-stabilised microRNA expression before batch correction.**

Principal component analysis was performed on variance-stabilised microRNA expression values. (A) Samples are coloured according to histotype: normal ovarian tissue, endometrioid ovarian carcinoma (EnOC), and ovarian clear cell carcinoma (OCCC). (B) The same samples are coloured according to dataset of origin and histotype, illustrating dataset-associated variation. The first and second principal components explain 24% and 11% of the total variance, respectively.
